# Novel three-dimensional coordination polymer of 2-(1,3,5-tri­aza-7-phospho­niatri­cyclo­[3.3.1.1^3,7^]decan-7-yl)ethanoic acid with silver(I) tetra­fluoro­borate

**DOI:** 10.1107/S2056989022000767

**Published:** 2022-02-01

**Authors:** Antal Udvardy, Ágnes Kathó, Gábor Papp, Ferenc Joó, Gyula Tamás Gál

**Affiliations:** a Department of Physical Chemistry, University of Debrecen, Debrecen, Hungary; b MTA-DE Redox and Homogeneous Catalytic Reaction Mechanisms Research Group, Department of Physical Chemistry, University of Debrecen, Debrecen, Hungary

**Keywords:** crystal structure, coordination polymer, silver, phosphabetaine, tetra­fluoro­borate

## Abstract

An Ag^I^-based coordination polymer (CP), namely, poly[[[μ_3_-2-(1,3,5-tri­aza-7-phospho­niatri­cyclo­[3.3.1.1^3,7^]decan-7-yl)ethano­ate-κ^4^ *N*:*N*′:*O*,*O*′]silver(I)] tetra­fluoro­borate], {[Ag(C_9_H_16_N_3_O_2_P)]BF_4_}_*n*_, was synthesized in an aqueous solution of zwitterionic 2-(1,3,5-tri­aza-7-phospho­niatri­cyclo­[3.3.1.1^3,7^]decan-7-yl)ethan­o­ate (**L**) and AgBF_4_ with exclusion of light at room temperature. The colourless and light-insensitive CP crystallized in the monoclinic space group *Cc*. The asymmetric unit consists of an Ag^I^ cation, the zwitterionic **L** ligand and a BF_4_^−^ counter-ion. Each Ag^I^ ion is coordinated by two carboxyl­ate oxygen atoms in a chelating coordination mode, as well as one of the nitro­gen atoms of two neighbouring **L** ligands. The crystal structure of the CP was classified as a unique three-dimensional arrangement. The CP was also characterized in aqueous solutions by multinuclear NMR and HRMS spectroscopies and elemental analysis.

## Abstract 2

A three-dimensional coordination polymer of 2-(1,3,5-tri­aza-7-phospho­niatri­cyclo­[3.3.1.1^3,7^]decan-7-yl)ethano­ate with silver(I) tetra­fluoro­borate was fully characterized.

## Chemical context

The architectures and anti­microbial properties of self-assembled silver-based coordination polymers (CPs) or MOFs (metal–organic frameworks), bridged by phosphaurotropines, have been widely studied (Guerriero *et al.*, 2018 ▸). According to our previous studies, the aqueous reaction of zwitterionic 2-(1,3,5-tri­aza-7-phospho­niatri­cyclo­[3.3.1.1^3,7^]decan-7-yl)ethan­o­ate (**L)** with Ag*X* (*X* = PF_6_, SO_3_C_6_H_4_CH_3_, SO_3_CF_3_) yielded various 1D Ag-based coordination polymers (Udvardy *et al.*, 2021 ▸). The architectures of these Ag^I^ complexes depend on their counter-ions and the position of the ligand, which contains both rigid and flexible mol­ecular moieties. 
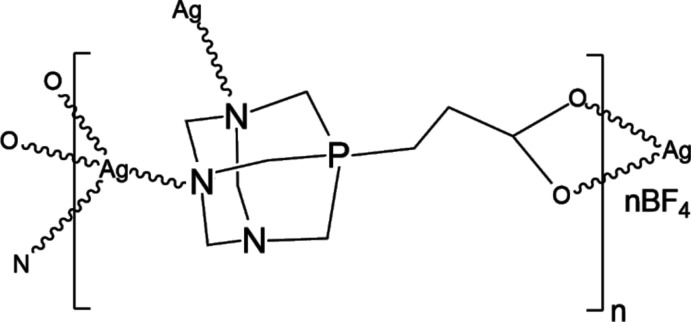


**Figure 1:**
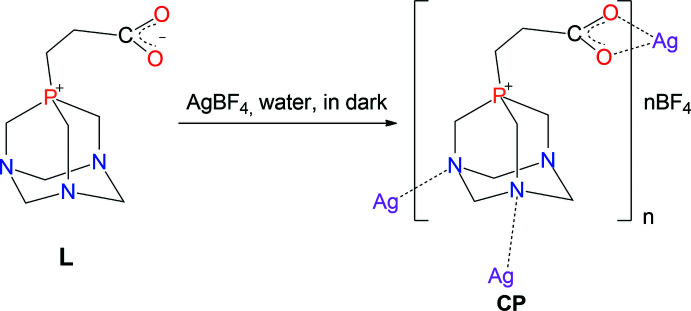
Schematic representation of the formation of the title compound.

Herein, we report the crystal structure of a CP prepared by the aqueous reaction of 2-(1,3,5-tri­aza-7-phospho­niatri­cyclo­[3.3.1.1^3,7^]decan-7-yl)ethano­ate and AgBF_4_ with the exclusion of light at 278 K (Fig. 1 ▸). The colourless crystals of the CP were isolated by filtration, dissolved in water and characterized by ^1^H-, ^13^C- and ^31^P-NMR spectroscopy, ESI mass spectrometry, as well as by elemental analysis.

The chemical shift of the phospho­rus atom in CP (δ = −37.5 ppm in D_2_O) was the same as that in the free ligand. Similar to the hexa­fluoro­phosphate, tosyl­ate (tos) and triflate (OTf) derivatives (Udvardy *et al.*, 2021 ▸), the ^1^H-NMR spectrum showed differences between the P^+^–CH_2_–N and N–CH_2_–N signals, which clearly indicated the coordination of the silver ions to the nitro­gen donor atoms of the **L** ligand.

The most intense ESI–MS signals of the CP (aqueous solution, positive ion mode) were observed at *m*/*z* = 252.0878 ([**L**+Na]^+^, C_9_H_16_N_3_NaO_2_P, calculated. 252.0872), 336.0026 ([**L**+Ag]^+^, C_9_H_16_N_3_AgO_2_P, calculated 336.0026), and 565.1009 ([2**L**+Ag]^+^, C_18_H_32_N_6_NaO_4_P_2_, calculated 565.1005). Similar ions were detected for the CP formed with AgPF_6_, AgSO_3_C_6_H_4_CH_3_, AgSO_3_CF_3_ and PTA in aqueous solutions.

## Structural commentary

**Figure 2:**
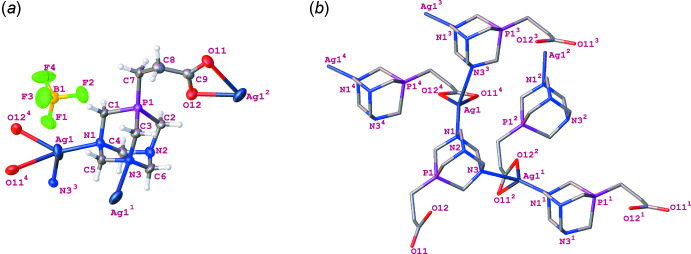
(*a*) A view of the title CP with atomic labels. Displacement ellipsoids are drawn at the 50% probability level. (*b*) The coordination architecture of the CP with atomic labels for the coordination sphere. Hydrogen atoms and BF_4_^−^ ions are omitted for clarity. [Symmetry codes: (1) *x*, −*y*, 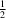 + *z*; (2) − 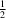 + *x*, − 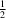 − *y*, 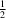 + *z*; (3) *x*, −*y*, − 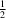 + *z*; (4) 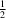 + *x*, − 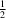 − *y*, − 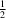 + *z*.]

The mol­ecular structure of the title compound is shown in Fig. 2 ▸. The CP crystallized in the monoclinic *Cc* space group. The asymmetric unit consists of a silver(I) cation, a zwitterionic **L** ligand and a BF_4_^−^counter-ion, in which the *N,N′,O,O′* coordination mode of the silver(I) ions creates a 3D coordination architecture (Fig. 2 ▸).

**Figure 3:**
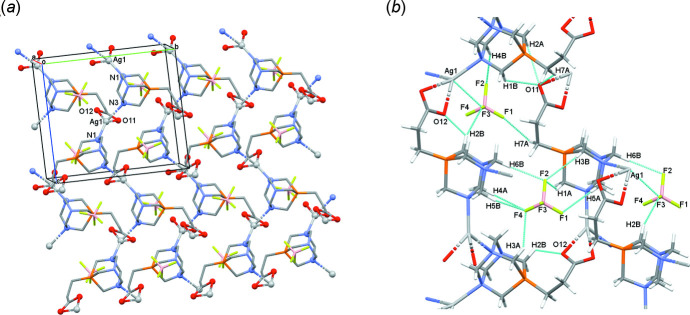
(*a*) Packing arrangement of the three-dimensional structure of the CP in the crystal viewed along the crystallographic *a* axis. The coordination sphere is labelled and highlighted by a ball-and-stick model. Hydrogen atoms are omitted for clarity. (*b*) Selected hydrogen-bond geometry in the CP showing the weak C—H⋯F and C—H⋯O secondary inter­actions, as well as the Ag1⋯F3 inter­action. For symmetry codes, see Table 1 ▸.

In the CP, the central Ag^+^ ion is coordinated by an **L** ligand *via* two carboxyl­ate oxygen atoms [Ag1^2^—O11 = 2.594 (9) Å and Ag1^2^—O12 = 2.298 (8) Å] and two nitro­gen atoms from two adjacent PTA moieties of **L** [Ag1—N1 = 2.225 (7) Å and Ag1^1^—N3 = 2.505 (7) Å]. The N1—Ag—N3^3^ and O11^4^—Ag—O12^4^ bond angles are 119.6 (3) and 52.9 (2)°, respectively. Selected bond lengths and bond angles are presented in the supporting information. The coordination geometry exhibits a distorted tetra­hedral shape (τ_4_ = 0.65 and τ_4_’ = 0.66; Yang *et al.*, 2007 ▸; Okuniewski *et al.*, 2015 ▸), in which the Ag^I^ ion is located at the centre. The space between the 3D polymer backbones is occupied by the BF_4_^−^ counter-ions (Fig. 3 ▸). The chemical composition was also determined by elemental analysis, which shows a good agreement with the SC-XRD results (see *Synthesis and crystallization*).

## Supra­molecular features

As a result of the lack of primary H-donor groups, no classical hydrogen bonds are found in the crystal structure of the title coordination polymer. The main inter­molecular inter­actions between the mol­ecules in the crystal are weak C—H⋯F and C—H⋯O type hydrogen bonds. The BF_4_^−^ anion is generally classified as a non-coordinating anion owing to its weak Lewis base properties (Grabowski, 2020 ▸). These secondary inter­actions play a major role in stabilizing the crystal lattice by connecting the mol­ecular units to each other, which results in a 3D coordination polymer. All of the fluorine atoms of a BF_4_^−^ counter-ion are connected to at least one C—H hydrogen atom by a weak C—H⋯F type hydrogen bond. The shortest C—H⋯F distance is found for the C2—H2*B*⋯F3 inter­action [C2⋯F3 = 3.183 (13) Å], where the F3 atom of the BF_4_^−^ counter-ion is also able to coordinate to the central Ag^+^ ion with a distance of 3.010 (11) Å (Fig. 3 ▸). This ionic attraction between the Ag^+^ and BF_4_^−^ ions is strong enough to affect the arrangement of part of the whole complex mol­ecule and form a bent 3D structure. In comparison, the value of the longest C—H⋯F distance is 3.417 (14) Å (C4—H4*B*⋯F2, Fig. 3 ▸) owing to the rigid PTA cage, which is unable to change its conformation. There are numerous examples in the literature of where the C—H⋯F distances were investigated in the presence of BF_4_^−^counter-ions [*i.e*. BIXBIT03 (Emge *et al.*, 1986 ▸) and SUXHID01 (Albinati *et al.*, 2010 ▸)]. In case of the bis­[μ_2_-1,1′-naphthalene-1,8-diyl-bis­(1*H*-pyrazole)]tris­(aceto­nitrile)­disilver(I) bis­(BF_4_) aceto­nitrile solvate structure (OGINOI; Liddle *et al.*, 2009 ▸), it was found that the typical C⋯F distances are between 3.179 (2) and 3.406 (3) Å, which shows a good agreement with our results. The carboxyl­ate oxygen atoms in the title CP are also able to form weak C—H⋯O type inter­actions with the C—H atoms of the complex mol­ecule. Their atomic distances can also be compared to the C—H⋯F secondary inter­actions. An intra­molecular hydrogen bond also helps to form a bent 3D mol­ecular structure for the CP [C2⋯O12 = 2.812 (12) Å]. For selected hydrogen-bond distances and angles see Fig. 3 ▸ *b* and Table 1 ▸. The considerably high calculated density (2.102 Mg m^−3^) and KPI (Kitaigorodskii packing index) of 74.2% (Spek, 2020 ▸) indicate the tight packing arrangement of the mol­ecules, resulting in no residual solvent-accessible voids in the crystal structure.

## Database survey

A survey of the Cambridge Structural Database (CSD version 5.42, Sept. 2021 update; Groom *et al.*, 2016 ▸) found zwitterionic 2-(1,3,5-tri­aza-7-phospho­niatri­cyclo­[3.3.1.1^3,7^]decan-7-yl)eth­ano­ate dihydrate (**L**) (SIJPOR; Tang *et al.*, 2007 ▸) and three 1D Ag-based coordination polymers containing **L**, *viz*. [Ag(μ_3_-**L**-κ^3^ *N*:*O*:*O*′)]_*n*_(PF_6_)_*n*_ (UPUCAM; Udvardy *et al.*, 2021 ▸), [Ag(OTf)(μ_3_-**L**-κ^3^ *N*:*O*:*O*′)]_*n*_ (UPUCIU; Udvardy *et al.*, 2021 ▸) and [Ag(tos)(μ_3_-**L**-κ^3^ *N*:*N*:*O*)]_*n*_·*n*H_2_O (UPUCEQ; Udvardy *et al.*, 2021 ▸). While in the cases of UPUCAM, UPUCIU and UPUCEQ only 1D polymers were obtained, in the title CP the Ag^I^ complex is able to form a 3D coordination polymer owing to the relatively small size of the BF_4_^−^ counter-ion, which is able to occupy a smaller space compared to the PF_6_^−^, triflate or tosyl­ate anions. These results show how a counter-ion can influence the packing arrangement and the coordination mode of an [(Ag**L**)*X*] type polymer.

## Synthesis and crystallization

Water-soluble PTA (Daigle, 1998 ▸) and 2-(1,3,5-tri­aza-7-phospho­niatri­cyclo­[3.3.1.1^3,7^]decan-7-yl)ethano­ate (**L**) (Tang *et al.*, 2007 ▸; Udvardy *et al.*, 2021 ▸) were prepared according to literature methods.

CP: With the exclusion of light, 4 mL aqueous solution containing 194.7 mg (1 mmol) AgBF_4_ was added to an aqueous solution (4 mL) of **L** (100 mg, 0.44 mmol). The reaction mixture was stored at 278 K. After two days, the CP was formed as colourless crystals, which were separated by filtration and dried. Yield (based on **L**) 112 mg, 60%. ^1^H NMR (360 MHz, D_2_O, 298 K) *δ* 4.73–4.37 (*m*, 12H, ^+^P–CH_2_–N, N–CH_2_–N), 2.58 (d*t*, *J* = 24, 7 Hz, 2H, P^+^–C*H*_2_–*C*H_2_–COO), 2.44–2.22 (*m*, 2H, P^+^–CH_2_–*CH*_2_–COO) ppm. ^13^C{^1^H} NMR (90 MHz, D_2_O, 298 K) *δ* 179.5 (*s*, *C*OO^−^), 71.5 (*d*, ^3^ *J*_PC_ = 8 Hz, N–*C*H_2_–N), 49.1 (*d*, ^1^ *J*_PC_ = 37 Hz, ^+^P-*C*H_2_–N), 29.0 (*d*, ^2^ *J*_PC_ = 7 Hz, P^+^–CH_2_–*C*H_2_–COO^−^), 18.5 (*d*, ^1^ *J*_PC_ = 35 Hz, P^+^–*C*H_2_–CH_2_–COO^−^) ppm. ^31^P{^1^H} NMR (145 MHz, D_2_O, 25 °C) *δ* −37.5 (*s*) ppm. Elemental analysis: C_9_H_16_AgBF_4_N_3_O_2_P (423.89): calculated C 25.05, H 3.80, N 9.91; found C 25.64, H 4.10, N 9.95.

## Refinement

Crystal data, data collection and structure refinement details are summarized in Table 2 ▸. All hydrogen atoms of the CP complex were positioned geometrically and refined using a riding model, with C—H = 0.97 Å and *U*_iso_(H) = 1.2*U*_eq_(C).

## Supplementary Material

Crystal structure: contains datablock(s) I. DOI: 10.1107/S2056989022000767/ex2052sup1.cif

Structure factors: contains datablock(s) I. DOI: 10.1107/S2056989022000767/ex2052Isup2.hkl

Supporting information file. DOI: 10.1107/S2056989022000767/ex2052Isup3.mol

Cover letter. DOI: 10.1107/S2056989022000767/ex2052sup4.pdf

Supporting information file. DOI: 10.1107/S2056989022000767/ex2052sup5.docx

Supporting information file. DOI: 10.1107/S2056989022000767/ex2052sup6.pdf

Cover letter. DOI: 10.1107/S2056989022000767/ex2052sup7.pdf

Supporting information file. DOI: 10.1107/S2056989022000767/ex2052sup8.pdf

CCDC reference: 2143743

Additional supporting information:  crystallographic information; 3D view; checkCIF report

## Figures and Tables

**Table 1:** Hydrogen-bond geometry (Å, °)

*D*—H⋯*A*	*D*—H	H⋯*A*	*D*⋯*A*	*D*—H⋯*A*
C1—H1*A*⋯F2	0.97	2.40	3.201 (13)	140
C1—H1*B*⋯O11^i^	0.97	2.49	3.213 (12)	131
C2—H2*A*⋯O11^i^	0.97	2.56	3.254 (12)	129
C2—H2*B*⋯F3^ii^	0.97	2.35	3.183 (13)	143
C2—H2*B*⋯O12	0.97	2.22	2.812 (12)	118
C3—H3*A*⋯F4^iii^	0.97	2.45	3.290 (15)	145
C3—H3*B*⋯F2	0.97	2.51	3.283 (12)	136
C4—H4*A*⋯F4^iv^	0.97	2.43	3.298 (12)	148
C4—H4*B*⋯F2^v^	0.97	2.51	3.417 (14)	155
C5—H5*A*⋯F1	0.97	2.49	3.370 (13)	151
C5—H5*B*⋯F4^iv^	0.97	2.54	3.373 (14)	144
C6—H6*B*⋯F2^iv^	0.97	2.34	3.314 (13)	177
C7—H7*A*⋯O11^i^	0.97	2.48	3.165 (13)	128
C7—H7*A*⋯F1^vi^	0.97	2.37	3.137 (12)	135

Symmetry codes: (i) 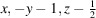 ; (ii) 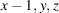 ; (iii) 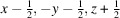 ; (iv) 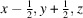 ; (v) 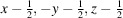 ; (vi) 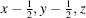 .

**Table 2:** Experimental details

Crystal data	
Chemical formula	[Ag(C_9_H_16_N_3_O_2_P)]BF_4_
*M*_r_	423.90
Crystal system, space group	Monoclinic, *C* *c*
Temperature (K)	293
*a*, *b*, *c* (Å)	10.116 (5), 12.186 (5), 10.979 (5)
β (°)	98.260 (5)
*V* (Å^3^)	1339.4 (11)
*Z*	4
Radiation type	Mo *K*α
μ (mm^−1^)	1.68
Crystal size (mm)	0.35 × 0.2 × 0.15

Data collection	
Diffractometer	Enraf–Nonius CAD-4
Absorption correction	ψ scan (North *et al.*, 1968 ▸)
*T*_min_, *T*_max_	0.558, 0.755
No. of measured, independent and observed [*I* > 2σ(*I*)] reflections	1358, 1313, 1299
*R*_int_	0.009
(sin θ/λ)_max_ (Å^−1^)	0.605

Refinement	
*R*[*F* ^2^ > 2σ(*F* ^2^)], *wR*(*F* ^2^), *S*	0.048, 0.123, 1.13
No. of reflections	1313
No. of parameters	190
No. of restraints	2
H-atom treatment	H-atom parameters constrained
Δρ_max_, Δρ_min_ (e Å^−3^)	1.20, −1.54
Absolute structure	Classical Flack method preferred over Parsons because s.u. lower
Absolute structure parameter	0.13 (6)

Computer programs: *MACH3/PC* (Enraf–Nonius, 1992 ▸), *PROFIT* (Streltsov & Zavodnik, 1989 ▸), *SIR97* (Burla *et al.*, 2007 ▸), *SHELXL* (Sheldrick, 2015 ▸), *OLEX2* (Dolomanov *et al.*, 2009 ▸) and *publCIF* (Westrip, 2010 ▸).

## supplementary crystallographic information

### Crystal data

**Table:**

[Ag(C_9_H_16_N_3_O_2_P)]BF_4_	*F*(000) = 840
*M**_r_* = 423.90	*D*_x_ = 2.102 Mg m^−^^3^
Monoclinic, *C**c*	Mo *K*α radiation, λ = 0.71073 Å
*a* = 10.116 (5) Å	Cell parameters from 25 reflections
*b* = 12.186 (5) Å	θ = 9.1–17.2°
*c* = 10.979 (5) Å	µ = 1.68 mm^−^^1^
β = 98.260 (5)°	*T* = 293 K
*V* = 1339.4 (11) Å^3^	Prism, colourless
*Z* = 4	0.35 × 0.2 × 0.15 mm

### Data collection

**Table:**

Enraf–Nonius CAD-4 diffractometer	*R*_int_ = 0.009
profiled ω/2θ scans	θ_max_ = 25.5°, θ_min_ = 3.1°
Absorption correction: ψ scan (North *et al.*, 1968)	*h* = 0→12
*T*_min_ = 0.558, *T*_max_ = 0.755	*k* = 0→14
1358 measured reflections	*l* = −13→13
1313 independent reflections	3 standard reflections every 184 reflections
1299 reflections with *I* > 2σ(*I*)	intensity decay: 2%

### Refinement

**Table:**

Refinement on *F*^2^	Hydrogen site location: inferred from neighbouring sites
Least-squares matrix: full	H-atom parameters constrained
*R*[*F*^2^ > 2σ(*F*^2^)] = 0.048	*w* = 1/[σ^2^(*F*_o_^2^) + (0.0873*P*)^2^ + 4.2725*P*] where *P* = (*F*_o_^2^ + 2*F*_c_^2^)/3
*wR*(*F*^2^) = 0.123	(Δ/σ)_max_ < 0.001
*S* = 1.13	Δρ_max_ = 1.20 e Å^−^^3^
1313 reflections	Δρ_min_ = −1.54 e Å^−^^3^
190 parameters	Absolute structure: Classical Flack method preferred over Parsons because s.u. lower
2 restraints	Absolute structure parameter: 0.13 (6)
Primary atom site location: structure-invariant direct methods	

### Special details

**Table:**

Geometry. All esds (except the esd in the dihedral angle between two l.s. planes) are estimated using the full covariance matrix. The cell esds are taken into account individually in the estimation of esds in distances, angles and torsion angles; correlations between esds in cell parameters are only used when they are defined by crystal symmetry. An approximate (isotropic) treatment of cell esds is used for estimating esds involving l.s. planes.

### Fractional atomic coordinates and isotropic or equivalent isotropic displacement parameters (Å^2^)

**Table:**

	*x*	*y*	*z*	*U*_iso_*/*U*_eq_	
C1	−0.0444 (9)	−0.2414 (7)	−0.2977 (8)	0.0266 (16)	
H1A	0.0509	−0.2557	−0.2849	0.032*	
H1B	−0.0820	−0.2737	−0.3758	0.032*	
C2	−0.2914 (9)	−0.2548 (7)	−0.2148 (9)	0.0302 (19)	
H2A	−0.3323	−0.2870	−0.2918	0.036*	
H2B	−0.3444	−0.2743	−0.1511	0.036*	
C3	−0.0546 (9)	−0.2182 (7)	−0.0445 (8)	0.0284 (17)	
H3A	−0.0975	−0.2365	0.0265	0.034*	
H3B	0.0408	−0.2298	−0.0232	0.034*	
C4	−0.2157 (10)	−0.1011 (7)	−0.3251 (9)	0.0317 (19)	
H4A	−0.2311	−0.0233	−0.3392	0.038*	
H4B	−0.2532	−0.1397	−0.3994	0.038*	
C5	−0.0139 (9)	−0.0704 (7)	−0.1840 (8)	0.0269 (16)	
H5A	0.0798	−0.0897	−0.1646	0.032*	
H5B	−0.0192	0.0087	−0.1934	0.032*	
C6	−0.2256 (10)	−0.0841 (8)	−0.1135 (10)	0.035 (2)	
H6A	−0.2706	−0.1113	−0.0472	0.042*	
H6B	−0.2418	−0.0057	−0.1203	0.042*	
C7	−0.0690 (9)	−0.4434 (7)	−0.1593 (9)	0.0311 (19)	
H7A	−0.1281	−0.4868	−0.2178	0.037*	
H7B	0.0201	−0.4492	−0.1815	0.037*	
C8	−0.0676 (11)	−0.4934 (8)	−0.0308 (9)	0.0340 (19)	
H8A	−0.0322	−0.5674	−0.0306	0.041*	
H8B	−0.0084	−0.4506	0.0285	0.041*	
C9	−0.2032 (10)	−0.4967 (8)	0.0078 (8)	0.0298 (18)	
N1	−0.0681 (7)	−0.1216 (6)	−0.3016 (6)	0.0253 (14)	
N2	−0.2837 (7)	−0.1355 (6)	−0.2263 (8)	0.0323 (17)	
N3	−0.0822 (8)	−0.1022 (6)	−0.0806 (7)	0.0278 (15)	
O11	−0.2377 (9)	−0.5691 (6)	0.0750 (8)	0.0444 (17)	
O12	−0.2793 (8)	−0.4175 (6)	−0.0319 (7)	0.0408 (16)	
P1	−0.1202 (2)	−0.30412 (17)	−0.1746 (2)	0.0234 (4)	
B1	0.3222 (13)	−0.2721 (10)	−0.1875 (11)	0.039 (2)	
Ag1	0.03866 (7)	−0.03737 (7)	−0.43859 (7)	0.0488 (3)	
F1	0.2885 (9)	−0.1691 (6)	−0.2226 (8)	0.062 (2)	
F2	0.2229 (8)	−0.3155 (7)	−0.1250 (9)	0.066 (2)	
F3	0.4361 (8)	−0.2745 (9)	−0.1032 (10)	0.082 (3)	
F4	0.3319 (16)	−0.3351 (7)	−0.2846 (9)	0.104 (4)	

### Atomic displacement parameters (Å^2^)

**Table:**

	*U*^11^	*U*^22^	*U*^33^	*U*^12^	*U*^13^	*U*^23^
C1	0.025 (4)	0.023 (4)	0.033 (4)	0.002 (3)	0.013 (3)	−0.004 (3)
C2	0.022 (4)	0.022 (4)	0.047 (5)	0.005 (3)	0.006 (4)	0.005 (4)
C3	0.029 (4)	0.027 (4)	0.029 (4)	0.007 (3)	0.003 (3)	0.001 (3)
C4	0.028 (5)	0.027 (4)	0.040 (5)	−0.001 (3)	0.001 (4)	0.008 (3)
C5	0.029 (4)	0.025 (4)	0.027 (4)	−0.004 (3)	0.008 (3)	−0.003 (3)
C6	0.031 (5)	0.028 (4)	0.049 (5)	0.010 (4)	0.019 (4)	−0.001 (4)
C7	0.026 (4)	0.029 (4)	0.042 (5)	−0.001 (3)	0.016 (4)	0.000 (4)
C8	0.040 (5)	0.031 (4)	0.030 (4)	0.004 (4)	0.001 (4)	0.009 (4)
C9	0.033 (5)	0.026 (4)	0.028 (4)	−0.001 (4)	−0.002 (3)	0.002 (4)
N1	0.023 (3)	0.025 (3)	0.027 (3)	−0.001 (3)	0.003 (3)	0.005 (3)
N2	0.023 (4)	0.027 (4)	0.048 (5)	0.001 (3)	0.008 (3)	0.005 (3)
N3	0.030 (4)	0.021 (3)	0.033 (4)	0.001 (3)	0.009 (3)	−0.002 (3)
O11	0.049 (4)	0.034 (3)	0.052 (4)	−0.002 (3)	0.013 (3)	0.016 (3)
O12	0.042 (4)	0.037 (3)	0.046 (4)	0.011 (3)	0.016 (3)	0.012 (3)
P1	0.0211 (10)	0.0193 (9)	0.0306 (10)	0.0024 (8)	0.0065 (8)	0.0009 (8)
B1	0.043 (7)	0.038 (6)	0.037 (5)	0.009 (5)	0.012 (5)	0.008 (5)
Ag1	0.0465 (4)	0.0623 (5)	0.0407 (4)	−0.0149 (4)	0.0167 (3)	0.0099 (4)
F1	0.059 (4)	0.037 (3)	0.090 (6)	0.000 (3)	0.007 (4)	0.016 (3)
F2	0.040 (4)	0.064 (5)	0.096 (6)	−0.004 (3)	0.016 (4)	0.024 (4)
F3	0.038 (4)	0.114 (8)	0.091 (6)	0.013 (5)	−0.003 (4)	0.020 (6)
F4	0.205 (14)	0.049 (4)	0.065 (5)	0.025 (6)	0.047 (7)	−0.003 (4)

### Geometric parameters (Å, º)

**Table:**

C1—H1A	0.9700	C6—N2	1.436 (14)
C1—H1B	0.9700	C6—N3	1.460 (12)
C1—N1	1.479 (11)	C7—H7A	0.9700
C1—P1	1.815 (9)	C7—H7B	0.9700
C2—H2A	0.9700	C7—C8	1.534 (13)
C2—H2B	0.9700	C7—P1	1.775 (9)
C2—N2	1.461 (11)	C8—H8A	0.9700
C2—P1	1.826 (9)	C8—H8B	0.9700
C3—H3A	0.9700	C8—C9	1.494 (15)
C3—H3B	0.9700	C9—O11	1.233 (12)
C3—N3	1.484 (11)	C9—O12	1.272 (12)
C3—P1	1.818 (9)	N1—Ag1	2.225 (7)
C4—H4A	0.9700	N3—Ag1^i^	2.505 (7)
C4—H4B	0.9700	O11—Ag1^ii^	2.594 (9)
C4—N1	1.499 (12)	O12—Ag1^ii^	2.298 (8)
C4—N2	1.428 (13)	B1—F1	1.343 (14)
C5—H5A	0.9700	B1—F2	1.399 (14)
C5—H5B	0.9700	B1—F3	1.370 (15)
C5—N1	1.467 (10)	B1—F4	1.328 (16)
C5—N3	1.464 (12)	Ag1—N3^iii^	2.505 (7)
C6—H6A	0.9700	Ag1—O11^iv^	2.594 (9)
C6—H6B	0.9700	Ag1—O12^iv^	2.298 (8)
			
H1A—C1—H1B	108.1	C7—C8—H8B	109.1
N1—C1—H1A	109.5	H8A—C8—H8B	107.8
N1—C1—H1B	109.5	C9—C8—C7	112.6 (8)
N1—C1—P1	110.7 (6)	C9—C8—H8A	109.1
P1—C1—H1A	109.5	C9—C8—H8B	109.1
P1—C1—H1B	109.5	O11—C9—C8	122.7 (9)
H2A—C2—H2B	108.6	O11—C9—O12	122.6 (10)
N2—C2—H2A	110.4	O12—C9—C8	114.7 (8)
N2—C2—H2B	110.4	C1—N1—C4	108.8 (7)
N2—C2—P1	106.7 (6)	C1—N1—Ag1	112.5 (5)
P1—C2—H2A	110.4	C4—N1—Ag1	112.0 (5)
P1—C2—H2B	110.4	C5—N1—C1	110.9 (6)
H3A—C3—H3B	108.5	C5—N1—C4	108.6 (7)
N3—C3—H3A	110.2	C5—N1—Ag1	104.0 (5)
N3—C3—H3B	110.2	C4—N2—C2	113.3 (7)
N3—C3—P1	107.8 (6)	C4—N2—C6	110.2 (8)
P1—C3—H3A	110.2	C6—N2—C2	112.3 (8)
P1—C3—H3B	110.2	C3—N3—Ag1^i^	115.1 (5)
H4A—C4—H4B	107.7	C5—N3—C3	111.6 (7)
N1—C4—H4A	108.9	C5—N3—Ag1^i^	93.4 (5)
N1—C4—H4B	108.9	C6—N3—C3	110.6 (7)
N2—C4—H4A	108.9	C6—N3—C5	109.4 (7)
N2—C4—H4B	108.9	C6—N3—Ag1^i^	115.4 (5)
N2—C4—N1	113.4 (7)	C9—O11—Ag1^ii^	85.8 (6)
H5A—C5—H5B	107.6	C9—O12—Ag1^ii^	98.7 (6)
N1—C5—H5A	108.7	C1—P1—C2	99.7 (4)
N1—C5—H5B	108.7	C1—P1—C3	101.4 (4)
N3—C5—H5A	108.7	C3—P1—C2	103.1 (4)
N3—C5—H5B	108.7	C7—P1—C1	108.9 (4)
N3—C5—N1	114.2 (7)	C7—P1—C2	126.3 (4)
H6A—C6—H6B	107.6	C7—P1—C3	114.0 (4)
N2—C6—H6A	108.6	F1—B1—F2	108.8 (9)
N2—C6—H6B	108.6	F1—B1—F3	111.6 (11)
N2—C6—N3	114.6 (7)	F3—B1—F2	104.7 (9)
N3—C6—H6A	108.6	F4—B1—F1	110.8 (10)
N3—C6—H6B	108.6	F4—B1—F2	108.4 (11)
H7A—C7—H7B	107.5	F4—B1—F3	112.2 (12)
C8—C7—H7A	108.4	N1—Ag1—N3^iii^	119.6 (3)
C8—C7—H7B	108.4	N1—Ag1—O11^iv^	134.1 (3)
C8—C7—P1	115.5 (7)	N1—Ag1—O12^iv^	133.5 (3)
P1—C7—H7A	108.4	N3^iii^—Ag1—O11^iv^	92.3 (3)
P1—C7—H7B	108.4	O12^iv^—Ag1—N3^iii^	103.6 (3)
C7—C8—H8A	109.1	O12^iv^—Ag1—O11^iv^	52.9 (2)
			
C7—C8—C9—O11	148.3 (10)	N2—C6—N3—C5	53.5 (10)
C7—C8—C9—O12	−33.2 (12)	N2—C6—N3—Ag1^i^	157.2 (6)
C8—C7—P1—C1	153.3 (7)	N3—C3—P1—C1	51.8 (7)
C8—C7—P1—C2	−88.5 (8)	N3—C3—P1—C2	−51.1 (7)
C8—C7—P1—C3	40.9 (8)	N3—C3—P1—C7	168.7 (6)
C8—C9—O11—Ag1^ii^	177.7 (9)	N3—C5—N1—C1	−66.7 (9)
C8—C9—O12—Ag1^ii^	−177.7 (7)	N3—C5—N1—C4	52.8 (9)
N1—C1—P1—C2	54.5 (7)	N3—C5—N1—Ag1	172.2 (6)
N1—C1—P1—C3	−51.1 (7)	N3—C6—N2—C2	71.6 (10)
N1—C1—P1—C7	−171.6 (6)	N3—C6—N2—C4	−55.7 (10)
N1—C4—N2—C2	−71.1 (10)	O11—C9—O12—Ag1^ii^	0.8 (11)
N1—C4—N2—C6	55.6 (10)	O12—C9—O11—Ag1^ii^	−0.7 (10)
N1—C5—N3—C3	70.1 (9)	P1—C1—N1—C4	−60.8 (8)
N1—C5—N3—C6	−52.7 (9)	P1—C1—N1—C5	58.5 (8)
N1—C5—N3—Ag1^i^	−171.2 (6)	P1—C1—N1—Ag1	174.5 (4)
N2—C2—P1—C1	−53.2 (7)	P1—C2—N2—C4	64.6 (9)
N2—C2—P1—C3	51.0 (7)	P1—C2—N2—C6	−61.0 (8)
N2—C2—P1—C7	−175.5 (6)	P1—C3—N3—C5	−62.5 (8)
N2—C4—N1—C1	66.6 (10)	P1—C3—N3—C6	59.5 (8)
N2—C4—N1—C5	−54.2 (9)	P1—C3—N3—Ag1^i^	−167.4 (3)
N2—C4—N1—Ag1	−168.4 (6)	P1—C7—C8—C9	62.9 (10)
N2—C6—N3—C3	−69.8 (10)		

Symmetry codes: (i) *x*, −*y*, *z*+1/2; (ii) *x*−1/2, −*y*−1/2, *z*+1/2; (iii) *x*, −*y*, *z*−1/2; (iv) *x*+1/2, −*y*−1/2, *z*−1/2.

### Hydrogen-bond geometry (Å, º)

**Table:**

*D*—H···*A*	*D*—H	H···*A*	*D*···*A*	*D*—H···*A*
C1—H1*A*···F2	0.97	2.40	3.201 (13)	140
C1—H1*B*···O11^v^	0.97	2.49	3.213 (12)	131
C2—H2*A*···O11^v^	0.97	2.56	3.254 (12)	129
C2—H2*B*···F3^vi^	0.97	2.35	3.183 (13)	143
C2—H2*B*···O12	0.97	2.22	2.812 (12)	118
C3—H3*A*···F4^ii^	0.97	2.45	3.290 (15)	145
C3—H3*B*···F2	0.97	2.51	3.283 (12)	136
C4—H4*A*···F4^vii^	0.97	2.43	3.298 (12)	148
C4—H4*B*···F2^viii^	0.97	2.51	3.417 (14)	155
C5—H5*A*···F1	0.97	2.49	3.370 (13)	151
C5—H5*B*···F4^vii^	0.97	2.54	3.373 (14)	144
C6—H6*B*···F2^vii^	0.97	2.34	3.314 (13)	177
C7—H7*A*···O11^v^	0.97	2.48	3.165 (13)	128
C7—H7*A*···F1^ix^	0.97	2.37	3.137 (12)	135

Symmetry codes: (ii) *x*−1/2, −*y*−1/2, *z*+1/2; (v) *x*, −*y*−1, *z*−1/2; (vi) *x*−1, *y*, *z*; (vii) *x*−1/2, *y*+1/2, *z*; (viii) *x*−1/2, −*y*−1/2, *z*−1/2; (ix) *x*−1/2, *y*−1/2, *z*.
