# Supplementary material for: Novel three-dimensional coordination polymer of 2-(1,3,5-tri­aza-7-phospho­niatri­cyclo­[3.3.1.13,7]decan-7-yl)ethanoic acid with silver(I) tetra­fluoro­borate
Source: Acta Crystallogr E Crystallogr Commun. 2022 Feb 1;78(Pt 3):251–4. doi: 10.1107/S2056989022000767 (PMC8900499; doi:10.1107/S2056989022000767)
Supplement: Supplementary file 5 [file e-78-00251-sup5.docx]

**Novel three-dimensional coordination polymer of 7-(2-carboxy-ethyl)-1,3,5-triaza-7-(phosphoniatricyclo)[3.3.1.1^3,7^]decane with silver(I)-tetraflouroborate**

**Antal Udvardy,* Ágnes Kathó, Gábor Papp, Ferenc Joó, Gyula Tamás Gál***

*University of Debrecen, Department of Physical Chemistry, P.O.Box 400, Debrecen, H-4002, Hungary*

Correspondence emails: udvardya@unideb.hu; gal.tamas@science.unideb.hu

**Keywords:** coordination polymer, silver, phosphabetaine, tetraflouroborate,

**Synopsis:** A three-dimensional coordination polymer of 7-(2-carboxy-ethyl)-1,3,5-triaza-7-(phosphoniatricyclo)[3.3.1.1^3,7^]decane with silver(I)-tetraflouroborate was fully characterized.

**Abstract**

An Ag(I)-based coordination polymer (**CP**) was synthesized in an aqueous solution of the zwitterionic 7-(2-carboxy-ethyl)-1,3,5-triaza-7-(phosphoniatricyclo)[3.3.1.1^3,7^]decane (**L**) and AgBF_4_ with exclusion of light at room temperature. The colourless and light insensitive **CP** crystallized in the monoclinic space group *Cc*.

The asymmetric unit consists of an Ag(I) cation, the zwitterionic **L** ligand and a $\text{BF}_{\text{4}}^{\text{–}}$ counter ion. Each Ag(I) ion is coordinated by two carboxylate oxygen atoms in chelate coordination mode, as well as one of the nitrogen atoms of two neighboring **L**. The crystal structure of **CP** was classified as a unique three-dimensional (3D) arrangement.

**CP** was also characterized in aqueous solutions by multinuclear NMR and HRMS spectroscopies and elemental analysis.


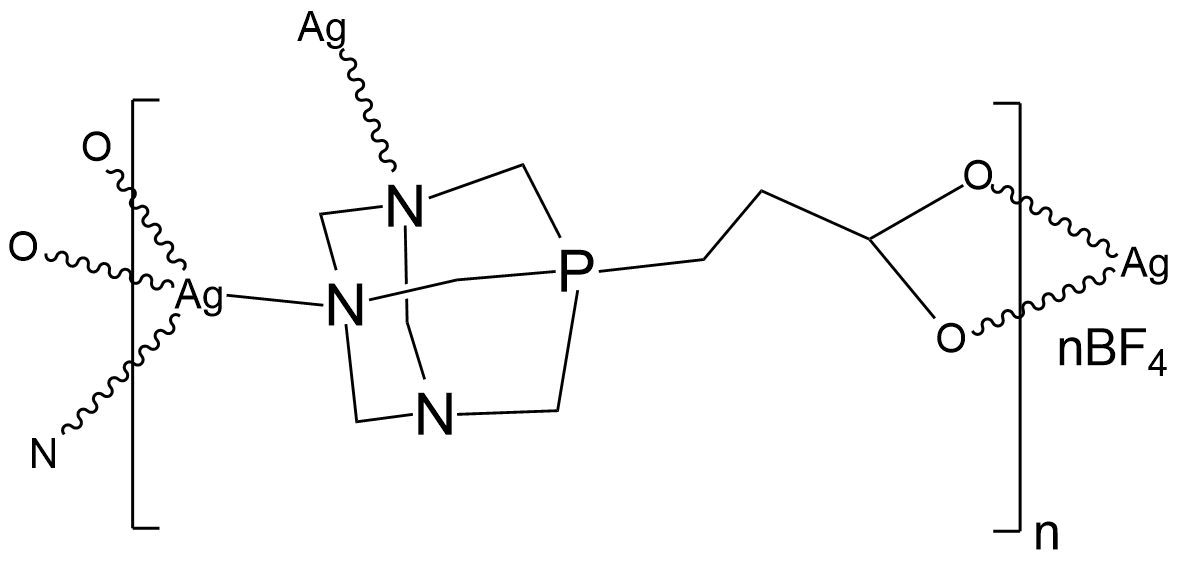


**1. Chemical context**

According to our previous studies, the aqueous reaction of zwitterionic 7-(2-carboxy-ethyl)-1,3,5-triaza-7-(phosphoniatricyclo)[3.3.1.1^3,7^]decane (**L)** with AgX (X=PF_6_, SO_3_C_6_H_4_CH_3_ SO_3_CF_3_) yielded various 1D Ag-based coordination polymers. [Udvardy *et al.,* 2021]. The architectures of these Ag(I)-complexes depend on their counter ions and the position of the ligand, which contain both rigid and flexible molecular moieties.

Herein, we report the crystal structure of **CP** prepared by aqueous reaction of 7-(2-carboxy-ethyl)-1,3,5-triaza-7-(phosphoniatricyclo)[3.3.1.1^3,7^]decane and AgBF_4_ with exclusion of light at 5°C (**Fig. 1**). The colourless crystals of **CP** were isolated by filtration, were dissolved in water and characterized by ^1^H-, ^13^C-, ^31^P-NMR spectroscopy, ESI mass spectrometry, as well as by elemental analysis.

The chemical shift of the phosphorus atom in **CP** (δ=–37.5 ppm in D_2_O) ~~–~~ was the same as that in the free ligand. Similar to the hexafluorophophate, tosylate (tos) and triflate (OTf) derivatives [Udvardy *et al.,* 2021], the ^1^H-NMR spectrum showed differences between the P^+^–CH_2_–N and N–CH_2_–N signals, which clearly indicated the coordination of silver ions to the nitrogen donor atoms of the **L**.

The most intense ESI-MS signals of **CP** (aqueous solution, positive ion mode) were observed at *m/z* 252.0878 ([**L**+Na]^+^, C_9_H_16_N_3_NaO_2_P, calculated. 252.0872), 336.0026 ([**L**+Ag]^+^, C_9_H_16_N_3_AgO_2_P, calculated 336.0026), and 565.1009 ([2**L**+Ag]^+^, C_18_H_32_N_6_NaO_4_P_2_, calculated 565.1005). Similar ions were detected for **CP** formed with AgPF_6_, AgSO_3_C_6_H_4_CH_3_, AgSO_3_CF_3_ and PTA in aqueous solutions.


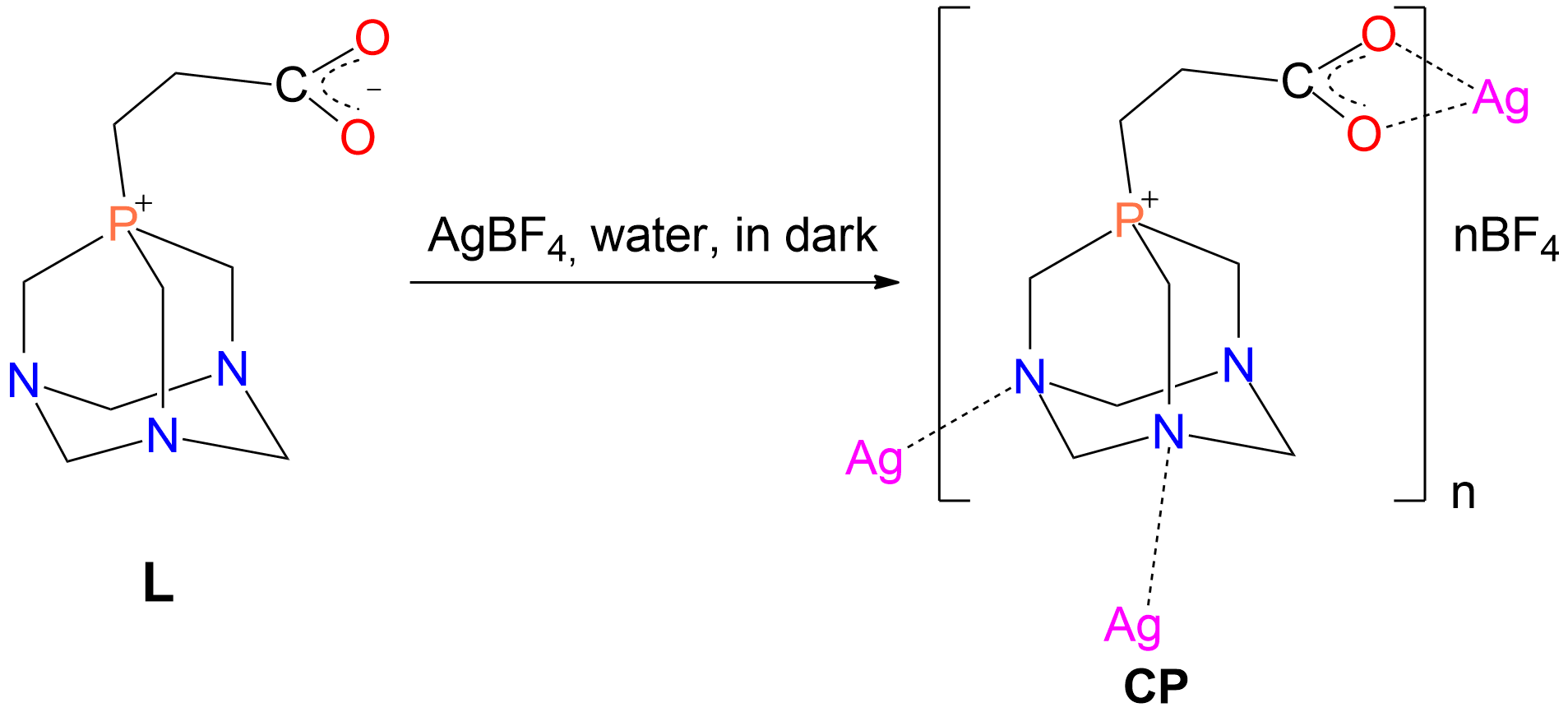


**Fig. 1.** Schematic diagram of formation of the title compound.

**2. Structural commentary**

The molecular structure of the title compound is shown in Fig. 2. **CP** crystallized in the monoclinic *Cc* space group. The asymmetric unit consists of a silver(I)-cation, a zwitterionic **L** ligand and a $\text{BF}_{\text{4}}^{\text{–}}$ counter ion, in which the N,N,O,O coordination mode of the silver(I)-ions creates a 3D coordination architecture (**Fig. 2.**)


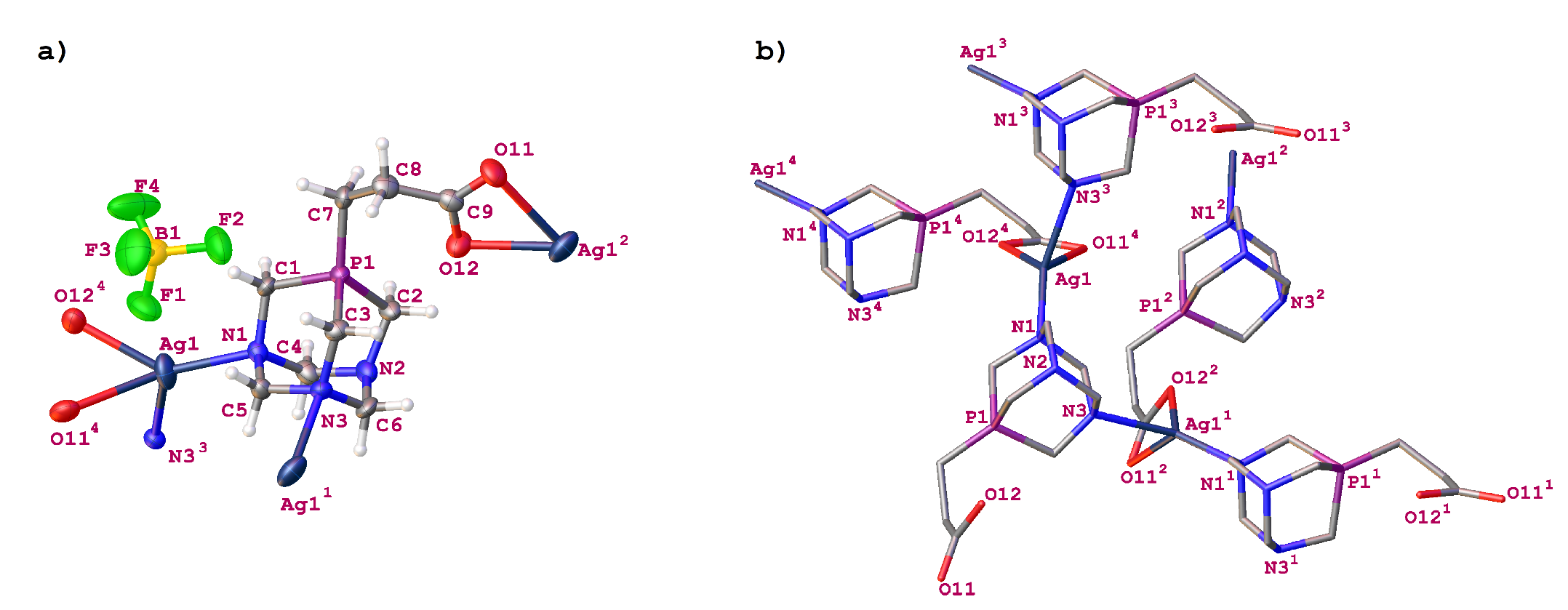


**Fig. 2.** (a) A view of the **CP** with the atomic labels. The atomic displacement ellipsoids are drawn at 50% probability level. (b) The coordination architecture of the **CP** with the atomic labels of the coordination sphere. Hydrogen atoms and $\text{BF}_{\text{4}}^{\text{–}}$ - ions are omitted for clarity. (Symmetry codes: ^1^+x,-y,1/2+z; ^2^-1/2+x,-1/2-y,1/2+z; ^3^+x,-y,-1/2+z; ^4^1/2+x,-1/2-y,-1/2+z)

In the **CP,** the central Ag^+^ ion is coordinated by an **L** ligand via two carboxylate oxygen atoms (Ag1^2^–O11=2.594(9) Å and Ag1^2^–O12=2.298(8) Å) and two nitrogen atoms from two adjacent PTA moieties of **L** (Ag1–N1=2.225(7) Å and Ag1^1^–N3=2.505(7) Å). The N1–Ag–N3^3^ and O11^4^–Ag–O12^4^ bond angles are 119.6(3)° and 52.9(2)°, respectively. The selected bond lengths and bond angles are presented in the Geometric parameters part in SI. The coordination geometry shows a distorted tetrahedral shape, in which the Ag(I) is located at the center. Among the 3D polymer backbones, the voids are occupied by $\text{BF}_{\text{4}}^{\text{–}}$ counter ions (**Fig. 2b**). The chemical composition was also determined by elemental analysis, which shows a good agreement with the SC-XRD results (See in Synthesis and crystallization chapter).

**3. Supramolecular features**

Because of the lack of primary H-donor groups no classic hydrogen bonds were found in the crystal lattice in the title coordination polymer. The main intermolecular interactions among the molecules in the crystal are only weak C–H…F and C–H…O type hydrogen bonds. The $\text{BF}_{\text{4}}^{\text{–}}$ anion is generally classified as a non-coordinating anion, owing to its weak Lewis base properties [Grabowski, 2020]. These secondary interactions play a major role in stabilizing the crystal lattice by connecting the molecular units to each other, which result in a 3D coordination polymer. All the fluorine atoms of a $\text{BF}_{\text{4}}^{\text{–}}$ counter ion are connected to at least one of a C–H hydrogen atom by a weak C–H…F type hydrogen bond. The shortest C–H…F distance is the C2–H2B…F3 interaction (C2–F3 = 3.183(13) Å), where the F3 atom of the BF_4_^-^ counter ion is also able to coordinate to the central Ag^+^ ion with a distance of 3.010(11) Å (**Fig. 3.**). This ionic attraction between the Ag^+^ and $\text{BF}_{\text{4}}^{\text{–}}$ is strong enough to arrange even a part of the whole complex molecule and form a bent 3D structure. In comparison, the value of the longest C–H…F distance is 3.417(14) Å (C4–H4B…F2, **Fig. 3.**) owing to the rigid PTA cage which is unable to change its conformation. There are numerous examples in the literature, where the C-H…F distances were investigated in the presence of $\text{BF}_{\text{4}}^{\text{–}}$ counter ions [i.e. BIXBIT03 and SUXHID01]. In case of the *bis*(μ_2_-1,1'-Naphthalene-1,8-diyl-bis(1H-pyrazole))-tris(acetonitrile)-di-silver(I)-bis(BF_4_) acetonitrile solvate structure (Ref Code: OGINOI) [Liddle *et al.*, 2009] it was found that the typical C…F distances are between 3.179(2) - 3.406(3) Å, which shows a good agreement with our results. The carboxylate oxygen atoms are also able to form weak C–H…O type interactions with the C–H atoms of the complex molecule. Their atomic distances can also be compared to the C–H…F secondary interactions. An intramolecular hydrogen bond also helps to form a bent 3D structure in crystal **CP** (C2–O12 = 2.812(12)Å). For the selected hydrogen bond distances and angles see **Fig. 3b**, and **Table 1**. The considerably high calculated density (2.102 Mg.m^-3^) and KPI (Kitaigorodskii packing index=74.2%) [Spek, 2020] indicate the tight packing arrangement of the molecules, resulting in no residual solvent-accessible voids in the crystal lattice.

**
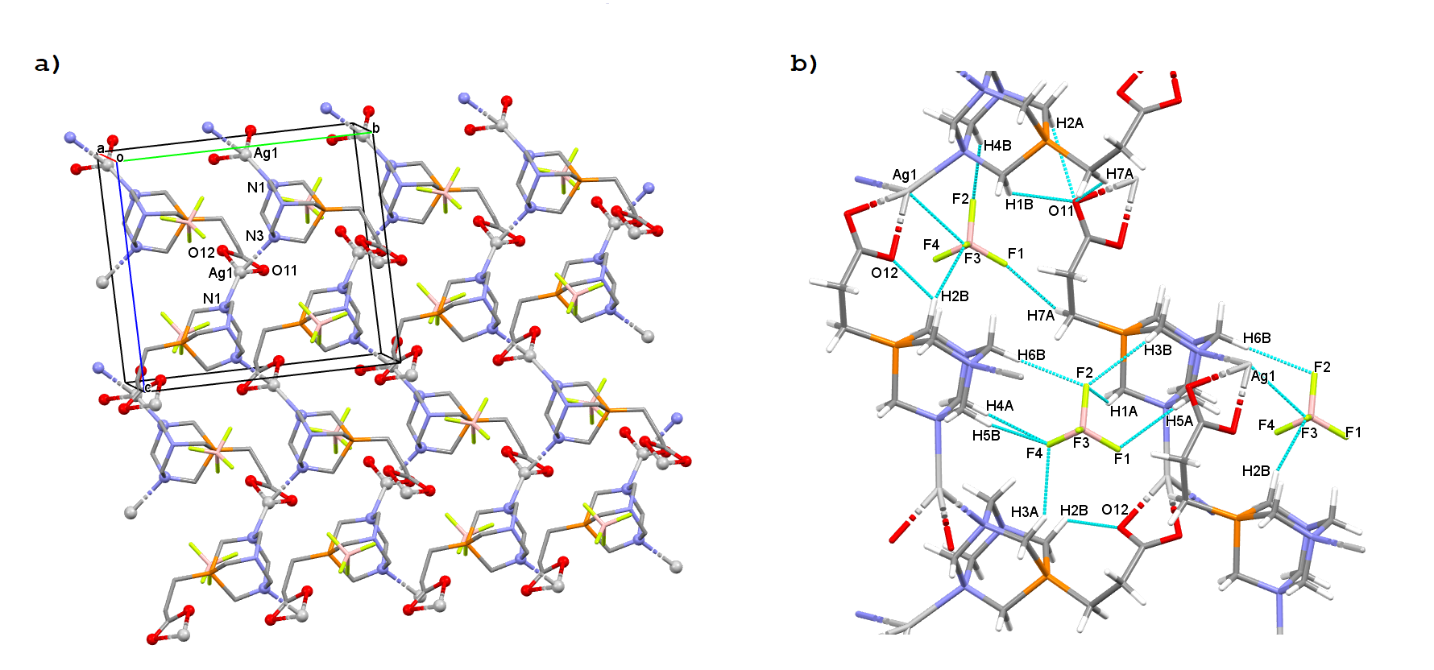
**

**Fig. 3:** (a) Packing arrangement of the three-dimensional structure of the **CP** crystal viewed from the crystallographic ‘***a***’ axes. The coordination sphere is labelled and highlighted by a ball and stick model. Hydrogen atoms are omitted for clarity. (b) Selected hydrogen bond geometry in **CP** showing the weak C–H…F and C–H…O secondary interactions, as well as the Ag1-F3 interaction. For symmetry codes see **Table 1.**

**Table 1.** Selected hydrogen bond distances (Å) and angles (^o^) in crystal **CP**

| *D*—H···*A* | *D*—H | H···*A* | *D*···*A* | *D*—H···*A* |
| --- | --- | --- | --- | --- |
| *C1*—*H1A*···*F2* | 0.97 | 2.40 | 3.201(13) | 140 |
| *C1*—H1B···*O11*^(i)^ | 0.97 | 2.49 | 3.213(12) | 131 |
| *C2*—*H2A*···*O11*^(i)^ | 0.97 | 2.56 | 3.254(12) | 129 |
| *C2*—*H2B*···*F3*^(ii)^ | 0.97 | 2.35 | 3.183(13) | 143 |
| *C2*—*H2B*···*O12* | 0.97 | 2.22 | 2.812(12) | 118 |
| *C3*—*H3A*···*F4*^(iii)^ | 0.97 | 2.45 | 3.290(15) | 145 |
| *C3*—*H3B*···*F2* | 0.97 | 2.51 | 3.283(12) | 136 |
| *C4*—*H4A*···*F4*^(iv)^ | 0.97 | 2.43 | 3.298(12) | 148 |
| *C4*—*H4B*···*F2*^(v)^ | 0.97 | 2.51 | 3.417(14) | 155 |
| *C5*—*H5A*···*F1* | 0.97 | 2.49 | 3.370(13) | 151 |
| *C5*—*H5B*···*F4*^(iv)^ | 0.97 | 2.54 | 3.373(14) | 144 |
| *C6*—*H6B*···*F2*^(iv)^ | 0.97 | 2.34 | 3.314(13) | 177 |
| *C7*—*H7A*···*O11*^ii)^ | 0.97 | 2.48 | 3.165(13) | 128 |
| *C7*—*H7A*···*F1*^(vi)^ | 0.97 | 2.37 | 3.137(12) | 135 |
|  |  |  |  |  |

*Symmetry codes: (i)* x, –1–y, –1/2+z; (ii) –1+x, y, z; (iii) –1/2+x, –1/2–y, 1/2+z; (iv) –1/2+x, 1/2+y, z;
(v) – 1/2+x, –1/2–y, –1/2+z; (vi) – 1/2+x, –1/2+y, z

**4. Database survey**

A survey of the Cambridge Structural Database (CSD version 5.42, May 2021 update; Groom et al., 2016) disclosed the zwitterionic 7-(2-carboxyethyl)-1,3,5-triaza-7-phosphoniatricyclo-[3.3.1.1^3,7^]decane dihydrate (**L**) (SIJPOR, [Tang *et al.*, 2007]) and three 1D Ag-based coordination polymers containing **L**. These are the [Ag(μ_3_-**L**-κ^3^*N:O:O’*)]_n_(PF_6_)_n_ (UPUCAM, [Udvardy *et al.,* 2021]), [Ag(OTf)(μ_3_-**L**-κ^3^*N:O:O’*)]_n_ (UPUCIU, [Udvardy *et al.,* 2021]) and the [Ag(tos)(μ_3_-**L**-κ^3^*N:N:O*)]_n_·nH_2_O (UPUCEQ, [Udvardy *et al.,* 2021]). While in the cases of UPUCAM, UPUCIU and UPUCEQ only 1D polymers were obtained, the Ag(I) complex – in case of the present **CP** – was able to form a 3D coordination polymer owing to the relatively small size of the $\text{BF}_{\text{4}}^{\text{–}}$ counter ion which is able to occupy a smaller space compared to the $\text{PF}_{\text{6}}^{\text{–}}$, triflate or tosylate anions. These results show how a counter ion can influence the packing arrangement and the coordination mode of an [(Ag-**L**)-X] type polymer compound.

**5. Synthesis and crystallization**

Water-soluble PTA [Daigle , 1998] and 7-(2-carboxy-ethyl)-1,3,5-triaza-7-(phosphoniatricyclo)-[3.3.1.1^3,7^]decane(**L**) [Tang *et al.*, 2007], [Udvardy *et al.,* 2021] were prepared according to literature methods.

**CP**: With the exclusion of light, 4 mL aqueous solution containing 194.7 mg (1 mmol) AgBF_4_ was added to an aqueous solution (4 mL) of **L** (100 mg, 0.44 mmol). The reaction mixture was stored at 5 °C. After two days, **CP** formed as colourless crystals, which were separated by filtration and dried. Yield (based on **L**) 112 mg, 60 %. ^1^H NMR (360 MHz, D_2_O, 25 °C) *δ* 4.73–4.37 (*m*, 12H, ^+^P–CH_2_–N, N–CH_2_–N), 2.58 (d*t*, *J*=24, 7 Hz, 2H, P^+^–C*H*_2_–CH_2_–COO), 2.44–2.22 (*m*, 2H, P^+^–CH_2_–C*H*_2_–COO) ppm. ^13^C{^1^H} NMR (90 MHz, D_2_O, 25 °C) δ 179.5 (*s*, COO^–^), 71.5 (d, ^3^J_PC_ = 8 Hz, N–CH_2_–N), 49.1 (*d*, ^1^J_PC_ = 37 Hz, ^+^P-CH_2_–N), 29.0 (d, ^2^J_PC_ = 7 Hz, P^+^–CH_2_–CH_2_–COO^–^), 18.5 (d, ^1^J_PC_ = 35 Hz, P^+^–CH_2_–CH_2_–COO^–^) ppm. ^31^P{^1^H} NMR (145 MHz, D_2_O, 25 °C) *δ* –37.5 (*s*) ppm. Elemental analysis: C_9_H_16_AgBF_4_N_3_O_2_P (423.89): calculated C 25.05, H 3.80, N 9.91; found C 25.64, H 4.10, N 9.95.

**6. Refinement**

Crystal data, data collection and details of the structure refinement are summarized in Table 2. All hydrogen atoms of the **CP** complex were positioned geometrically and refined using a riding model, with C—H = 0.97 Å.

**Acknowledgements**

The authors thank Ms Cynthia Nóra Nagy (University of Debrecen) for HRMS, Dr. Attila Kiss for the elemental analysis measurements. We are grateful to Dr. Attila Bényei (University of Debrecen) for recording the diffraction data.

**Funding information**

The financial support of the Hungarian National Research, Development and Innovation Office (FK-128333) is greatly acknowledged. Project no. TKP2020-NKA-04 has been implemented with the support provided from the National Research, Development and Innovation Fund of Hungary, financed under the 2020-4.1.1-TKP2020 funding scheme. The research was supported by the EU and co-financed by the European Regional Development Fund under the projects GINOP-2.3.3-15-2016-00004 and GINOP 2.3.2-15-2016-00008.

**References**

Burla, M. C., Caliandro, R., Camalli, M., Carrozzini, B., Cascarano, G. L., De Caro, L., Giacovazzo, C., Polidori, G., Siliqi, D. & Spagna, R. (2007). *J. Appl. Cryst*. **40**, 609–613.

Daigle, D. J. (1998). *Inorg. Synth*. **32**, 40–45.

Dolomanov, O. V., Bourhis, L. J., Gildea, R. J., Howard, J. A. K. & Puschmann, H. (2009). *J. Appl. Cryst.* **42**, 339–341.

Grabowski, S. J. (2020). *Crystals*, **10**, 460.

Groom, C. R., Bruno, I. J., Lightfoot, M. P. & Ward, S. C. (2016). *Acta Cryst*. **B72**, 171–179.

Liddle, B. L., Hall, D., Lindeman, S. J., Smith, M. D. & Gardinier, J. R. (2009). *Inorg. Chem.* **48**, 8404–8414.

MACH3/PC Software: Enraf-Nonius (1992). *CAD-4 Software* (or *CAD-4 EXPRESS*). Enraf-Nonius, Delft, The Netherlands.

North, A. C. T., Phillips, D. C. & Mathews, F. S. (1968). *Acta. Cryst.* **A24**, 351–359.

Sheldrick, G. M. (2015). *Acta Cryst.* **C71**, 3–8.

Spek, A. L. (2020). *Acta Cryst.* **E76**, 1–11.

Streltsov, V. A. & Zavodnik, V. E. (1989). *Sov. Phys. Crystallogr.* **34**, 824–828.

Tang, X., Zhang, B., He, Z., Gao, R. & He, Z. (2007). *Adv. Synth. Catal.* **349**, 2007–2017.

Udvardy, A., Szolnoki, Cs. T., Kováts, É., Nyul, D., Gál, Gy. T., Papp, G., Joó, F. & Kathó, Á. (2021). *Inorg. Chim. Act.* **520**, 120299.

Westrip, S. P. (2010). *J. Appl. Cryst.* **43**, 920–925.
