# Supplementary material for: Novel three-dimensional coordination polymer of 2-(1,3,5-tri­aza-7-phospho­niatri­cyclo­[3.3.1.13,7]decan-7-yl)ethanoic acid with silver(I) tetra­fluoro­borate
Source: Acta Crystallogr E Crystallogr Commun. 2022 Feb 1;78(Pt 3):251–4. doi: 10.1107/S2056989022000767 (PMC8900499; doi:10.1107/S2056989022000767)
Supplement: Supplementary file 6 [file e-78-00251-sup6.pdf]

Novel three-dimensional coordination polymer of 7-(2-carboxy-ethyl)-1,3,5-tri–aza-7-(phosphoniatricyclo)[3.3.1.1^{3,7}]decane with silver(I)-tetraflouroborate

Antal Udvardy,[a]‡ Agnes Katho,[a] Gabor Papp,[a] Ferenc Joo[ab] and Gyula Tamas Gal[a]*‡

[a]Department of Physical Chemistry, University of Debrecen, Debrecen, Hungary, and [b]MTA-DE Redox and Homogeneous Catalytic Reaction Mechanisms Research Group, Department of Physical Chemistry, University of Debrecen, Debrecen, Hungary

Correspondence email: gal.tamas@science.unideb.hu;_udvardya@unideb.hu

‡ Correspondence author

Abstract

An Ag(I)-based coordination polymer (**CP**) was synthesized in an aqueous solution of the zwitterionic 7-(2-carboxy-ethyl)-1,3,5-triaza-7-(phosphoniatricyclo)[3.3.1.1^{3,7}]decane (**L**) and $AgBF_4$ with exclusion of light at room temperature. The colourless and light insensitive **CP** crystallized in the monoclinic space group *Cc*.The asymmetric unit consists of an Ag(I) cation, the zwitterionic **L** ligand and a BF_4^- counter ion. Each Ag(I) ion is coordinated by two carboxylate oxygen atoms in chelate coordination mode, as well as one of the nitrogen atoms of two neighboring **L**. The crystal structure of **CP** was classified as a unique three-dimensional (3D) arrangement.**CP** was also characterized in aqueous solutions by multinuclear NMR and HRMS spectroscopies and elemental analysis.

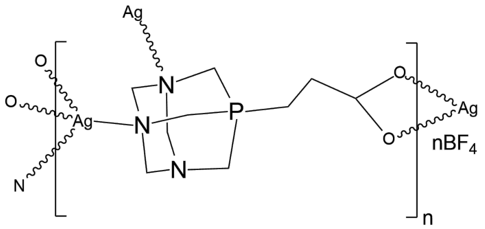

1. Chemical context

According to our previous studies, the aqueous reaction of zwitterionic 7-(2-carboxy-ethyl)-1,3,5-triaza-7-(phosphoniatricyclo)[3.3.1.1^{3,7}]decane (**L)** with AgX (X=PF_6, $SO_3C_6H_4CH_3$, SO_3CF_3) yielded various 1D Ag-based coordination polymers [Udvardy *et al.*, 2021]. The architectures of these Ag(I)-complexes depend on their counter ions and the position of the ligand, which contain both rigid and flexible molecular moieties.

Herein, we report the crystal structure of **CP** prepared by aqueous reaction of 7-(2-carboxy-ethyl)-1,3,5-triaza-7-(phosphoniatricyclo)[3.3.1.1^{3,7}]decane and $AgBF_4$ with exclusion of light at 5°C (**Fig. 1**). The colourless crystals of **CP** were isolated by filtration, were dissolved in water and characterized by ^{1}H-, ^{13}C-, ^{31}P-NMR spectroscopy, ESI mass spectrometry, as well as by elemental analysis.

The chemical shift of the phosphorus atom in **CP** (δ=–37.5 ppm in D_2O) – was the same as that in the free ligand. Similar to the hexafluorophophate, tosylate (tos) and triflate (OTf) derivatives [Udvardy *et al.*, 2021], the ^{1}H-NMR spectrum showed differences between the P^+–CH_2–N and N–CH_2–N signals, which clearly indicated the coordination of silver ions to the nitrogen donor atoms of the **L**.

The most intense ESI-MS signals of **CP** (aqueous solution, positive ion mode) were observed at *m/z* 252.0878 ([**L**+Na]$^+$, $C_9H_{16}N_3NaO_2P$, calculated. 252.0872), 336.0026 ([**L**+Ag]$^+$, $C_9H_{16}N_3AgO_2P$, calculated 336.0026), and 565.1009 ([2**L**+Ag]$^+$, $C_{18}H_{32}N_6NaO_4P_2$, calculated 565.1005). Similar ions were detected for **CP** formed with $AgPF_6$, $AgSO_3C_6H_4CH_3$, $AgSO_3CF_3$ and PTA in aqueous solutions.

Figure 1

2. Structural commentary

The molecular structure of the title compound is shown in **Fig. 2**. **CP** crystallized in the monoclinic *Cc* space group. The asymmetric unit consists of a silver(I)-cation, a zwitterionic **L** ligand and a BF_4^-counter ion, in which the N,N,O,O coordination mode of the silver(I)-ions creates a 3D coordination architecture (**Fig. 2**.)

Figure 2

In the **CP**, the central Ag^+ ion is coordinated by an **L** ligand via two carboxylate oxygen atoms ($Ag1^2$–O11=2.594 (9) Å and $Ag1^2$–O12=2.298 (8) Å) and two nitrogen atoms from two adjacent PTA moieties of **L** (Ag1–N1=2.225 (7) Å and $Ag1^1$–N3=2.505 (7) Å). The N1–Ag–$N3^3$ and $O11^4$–Ag–$O12^4$ bond angles are 119.6 (3)° and 52.9 (2)°, respectively. The selected bond lengths and bond angles are presented in the Geometric parameters part in SI. The coordination geometry shows a distorted tetrahedral shape, in which the Ag(I) is located at the center. Among the 3D polymer backbones, the voids are occupied by BF_4^- counter ions (**Fig. 2b**). The chemical composition was also determined by elemental analysis, which shows a good agreement with the SC-XRD results (see in the Synthesis and crystallization chapter).

3. Supramolecular features

Because of the lack of primary H-donor groups no classic hydrogen bonds were found in the crystal lattice in the title coordination polymer. The main intermolecular interactions among the molecules in the crystal are only weak C–H···F and C–H···O type hydrogen bonds. The BF_4^- anion is generally classified as a non-coordinating anion, owing to its weak Lewis base properties [Grabowski, 2020]. These secondary interactions play a major role in stabilizing the crystal lattice by connecting the molecular units to each other, which result in a 3D coordination polymer. All the fluorine atoms of a BF_4^- counter ion are connected to at least one of a C–H hydrogen atom by a weak C–H···F type hydrogen bond. The shortest C–H···F distance is the C2–H2B···F3 interaction (C2–F3 = 3.183 (13) Å), where the F3 atom of the BF_4^- counter ion is also able to coordinate to the central Ag^+ ion with a distance of 3.010 (11) Å (**Fig. 3.**). This ionic attraction between the Ag^+ and BF_4^- is strong enough to arrange even a part of the whole complex molecule and form a bent 3D structure. In comparison, the value of the longest C–H···F distance is 3.417 (14) Å (C4–H4B···F2, **Fig. 3**) owing to the rigid PTA cage which is unable to change its conformation. There are numerous examples in the literature, where the C—H···F distances were investigated in the presence of BF_4^-counter ions [i.e. BIXBIT03 and SUXHID01]. In case of the *bis*(μ_2-1,1'-Naphthalene-1,8-diyl-bis(1H-pyrazole))-tris(acetonitrile)-di-silver(I)-bis(BF_4) acetonitrile solvate structure (Ref Code: OGINOI) [Liddle *et al.*, 2009] it was found that the typical C···F distances are between 3.179 (2) - 3.406 (3) Å, which shows a good agreement with our results. The carboxylate oxygen atoms are also able to form weak C–H···O type interactions with the C–H atoms of the complex molecule. Their atomic distances can also be compared to the C–H···F secondary interactions. An intramolecular hydrogen bond also helps to form a bent 3D structure in crystal **CP** (C2–O12 = 2.812 (12)Å). For the selected hydrogen bond distances and angles see **Fig. 3b**, and **Table 1**. The considerably high calculated density (2.102 $Mg.m^{-3}$) and KPI (Kitaigorodskii packing index=74.2%) [Spek, 2020] indicate the tight packing arrangement of the molecules, resulting in no residual solvent-accessible voids in the crystal lattice.

Figure 3

4. Database survey

A survey of the Cambridge Structural Database (CSD version 5.42, Sept. 2021 update; [Groom *et al.*, 2016]) disclosed the zwitterionic 7-(2-carboxyethyl)-1,3,5-triaza-7-phosphoniatricyclo[3.3.1.1^{3,7}]decane dihydrate (**L**) (SIJPOR, [Tang *et al.*, 2007]) and three 1D Ag-based coordination polymers containing **L**. These are the [Ag(μ_3-**L**-κ^3*N:O:O'*)]$_n$(PF_6)$_n$ (UPUCAM, [Udvardy *et al.*, 2021]), [Ag(OTf)(μ_3-**L**-κ^3*N:O:O'*)]$_n$ (UPUCIU, [Udvardy *et al.*., 2021]) and the [Ag(tos)(μ_3-**L**-κ^3*N:N:O*)]$_n$·nH_2O (UPUCEQ, [Udvardy *et al.*, 2021]). While in the cases of UPUCAM, UPUCIU and UPUCEQ only 1D polymers were obtained, the Ag(I) complex – in case of the present **CP** – was able to form a 3D coordination polymer owing to the relatively small size of the BF_4^- counter ion which is able to occupy a smaller space compared to the PF_6^-, triflate or tosylate anions. These results show how a counter ion can influence the packing arrangement and the coordination mode of an [(Ag-**L**)-X] type polymer compound.

5. Synthesis and crystallization

Water-soluble PTA [Daigle, 1998] and 7-(2-carboxy-ethyl)-1,3,5-triaza-7-(phosphoniatricyclo)-[3.3.1.1^{3,7}]decane(**L**) [Tang *et al.*, 2007], [Udvardy *et al.*, 2021] were prepared according to literature methods.

CP: With the exclusion of light, 4 mL aqueous solution containing 194.7 mg (1 mmol) $AgBF_4$ was added to an aqueous solution (4 mL) of **L** (100 mg, 0.44 mmol). The reaction mixture was stored at 5 °C. After two days, **CP** was formed as colourless crystals, which were separated by filtration and dried. Yield (based on **L**) 112 mg, 60 %. ^{1}H NMR (360 MHz, D_2O, 25 °C) δ 4.73–4.37 (*m*, 12H, $^+$P–CH_2–N, N–CH_2–N), 2.58 (d*t*, *J*=24, 7 Hz, 2H, P$^+$–CH_2–CH_2–COO), 2.44–2.22 (*m*, 2H, P$^+$–CH_2–CH_2–COO) ppm. ^{13}C{^{1}H} NMR (90 MHz, D_2O, 25 °C) δ 179.5 (*s*, *C*OO$^-$), 71.5 (d, $^3J_{PC}$ = 8 Hz, N–*C*H_2–N), 49.1 (*d*, $^1J_{PC}$ = 37 Hz, $^+$P-*C*H_2–N), 29.0 (*d*, $^2J_{PC}$ = 7 Hz, P$^+$–CH_2–*C*H_2–COO$^-$), 18.5 (*d*, $^1J_{PC}$ = 35 Hz, P$^+$–*C*H_2–CH_2–COO$^-$) ppm. ^{31}P{^{1}H} NMR (145 MHz, D_2O, 25 °C) δ –37.5 (*s*) ppm. Elemental analysis: $C_9H_{16}AgBF_4N_3O_2P$ (423.89): calculated C 25.05, H 3.80, N 9.91; found C 25.64, H 4.10, N 9.95.

6. Refinement

Crystal data, data collection and structure refinement details are summarized in **Table 2**. All hydrogen atoms of the **CP** complex were positioned geometrically and refined using a riding model, with C—H = 0.97 Å.

Table 1

Table 1. Selected hydrogen bond distances (Å) and angles (°) in crystal CP

D—H···A	D—H	H···A	D···A	D—H···A
C1—H1A···F2	0.97	2.40	3.201 (13)	140
C1—H1B···O11$^{(i)}$	0.97	2.49	3.213 (12)	131
C2—H2A···O11$^{(i)}$	0.97	2.56	3.254 (12)	129
C2—H2B···F3$^{(ii)}$	0.97	2.35	3.183 (13)	143
C2—H2B···O12	0.97	2.22	2.812 (12)	118
C3—H3A···F4$^{(iii)}$	0.97	2.45	3.290 (15)	145
C3—H3B···F2	0.97	2.51	3.283 (12)	136
C4—H4A···F4$^{(iv)}$	0.97	2.43	3.298 (12)	148
C4—H4B···F2$^{(v)}$	0.97	2.51	3.417 (14)	155
C5—H5A···F1	0.97	2.49	3.370 (13)	151
C5—H5B···F4$^{(iv)}$	0.97	2.54	3.373 (14)	144
C6—H6B···F2$^{(iv)}$	0.97	2.34	3.314 (13)	177
C7—H7A···O11$^{(ii)}$	0.97	2.48	3.165 (13)	128
C7—H7A···F1$^{(vi)}$	0.97	2.37	3.137 (12)	135

Symmetry codes: (i) x, −1−y, −1/2+z; (ii) −1+x, y, z; (iii) −1/2+x, −1/2−y, 1/2+z; (iv) −1/2+x, 1/2+y, z; (v) − 1/2+x, −1/2−y, −1/2+z; (vi) − 1/2+x, −1/2+y, z

Table 2

Experimental details

Crystal data	
Chemical formula	$C_9H_{16}AgN_3O_2P\cdot BF_4$
M_r	423.90
Crystal system, space group	Monoclinic, *Cc*
Temperature (K)	293
a, *b*, *c* (Å)	10.116 (5), 12.186 (5), 10.979 (5)
β (°)	98.260 (5)
V (Å^3)	1339.4 (11)
Z	4
Radiation type	Mo *K*α
μ (mm^{-1})	1.68
Crystal size (mm)	0.35 × 0.2 × 0.15
Data collection	
Diffractometer	Enraf Nonius CAD4
Absorption correction	ψ scan<br>North A.C.T., Phillips D.C. & Mathews F.S. (1968) Acta. Cryst. A24, 351-359 Number of ψ scan sets used was 3 Theta correction was applied. Averaged transmission function was used. Fourier smoothing - Window value 5
T_{min}, T_{max}	0.558, 0.755
No. of measured, independent and observed [$I > 2\sigma(I)$] reflections	1358, 1313, 1299
R_{int}	0.009
$(\sin \theta/\lambda)_{max}$ (Å^{-1})	0.605
Refinement	
$R[F^2 > 2\sigma(F^2)]$, $wR(F^2)$, S	0.048, 0.123, 1.13
No. of reflections	1313
No. of parameters	190
No. of restraints	2
H-atom treatment	H-atom parameters constrained
$\Delta\rho_{max}$, $\Delta\rho_{min}$ (e Å^{-3})	1.20, −1.54
Absolute structure	Classical Flack method preferred over Parsons because s.u. lower.
Absolute structure parameter	0.13 (6)

Computer programs: MACH3/PC (Enraf Nonius, 1992), PROFIT (Streltsov & Zavodnik, 1989), *SIR97* (Burla *et al.*, 2007), *SHELXL* (Sheldrick, 2015), Olex2 1.3 (Dolomanov *et al.*, 2009), *publCIF* (Westrip, 2010).

Acknowledgements

The authors thank Ms Cynthia Nóra Nagy (University of Debrecen) for HRMS, Dr. Attila Kiss for the elemental analysis measurements. We are grateful to Dr. Attila Bényei (University of Debrecen) for recording the diffraction data.

The financial support of the Hungarian National Research, Development and Innovation Office (FK-128333) is greatly acknowledged. Project no. TKP2020-NKA-04 has been implemented with the support provided from the National Research, Development and Innovation Fund of Hungary, financed under the 2020-4.1.1-TKP2020 funding scheme. The

research was supported by the EU and co-financed by the European Regional Development Fund under the projects GINOP-2.3.3-15-2016-00004 and GINOP 2.3.2-15-2016-00008.

Funding information

Funding for this research was provided by: Hungarian National Research, Development and Innovation Office (contract No. FK-128333); Development and Innovation Fund of Hungary (contract No. 2020-4.1.1-TKP2020, Project no. TKP2020-NKA-04); European Regional Development Fund (contract No. GINOP-2.3.3-15-2016-00004; contract No. GINOP 2.3.2-15-2016-00008).

References

Burla, M. C., Caliandro, R., Camalli, M., Carrozzini, B., Cascarano, G. L., De Caro, L., Giacovazzo, C., Polidori, G., Siliqi, D. & Spagna, R. (2007). *J. Appl. Cryst*. **40**, 609–613.

Daigle, D. J. (1998). *Inorg. Synth*. **32**, 40–45.

Dolomanov, O. V., Bourhis, L. J., Gildea, R. J., Howard, J. A. K. & Puschmann, H. (2009). *J. Appl. Cryst.* **42**, 339–341.

Grabowski, S. J. (2020). *Crystals*, **10**, 460.

Groom, C. R., Bruno, I. J., Lightfoot, M. P. & Ward, S. C. (2016). *Acta Cryst*. **B72**, 171–179.

Liddle, B. L., Hall, D., Lindeman, S. J., Smith, M. D. & Gardinier, J. R. (2009). *Inorg. Chem.* **48**, 8404–8414.

MACH3/PC Software: Enraf-Nonius (1992). *CAD-4 Software* (or *CAD-4 EXPRESS*). Enraf-Nonius, Delft, The Netherlands.

North, A. C. T., Phillips, D. C. & Mathews, F. S. (1968). *Acta. Cryst.* **A24**, 351–359.

Sheldrick, G. M. (2015). *Acta Cryst.* **C71**, 3–8.

Spek, A. L. (2020). *Acta Cryst.* **E76**, 1–11.

Streltsov, V. A. & Zavodnik, V. E. (1989). *Sov. Phys. Crystallogr.* **34**, 824–828.

Tang, X., Zhang, B., He, Z., Gao, R. & He, Z. (2007). *Adv. Synth. Catal.* **349**, 2007–2017.

Udvardy, A., Szolnoki, Cs. T., Kováts, É., Nyul, D., Gál, Gy. T., Papp, G., Joó, F. & Kathó, Á. (2021). *Inorg. Chim. Act.* **520**, 120299.

Westrip, S. P. (2010). *J. Appl. Cryst.* **43**, 920–925.

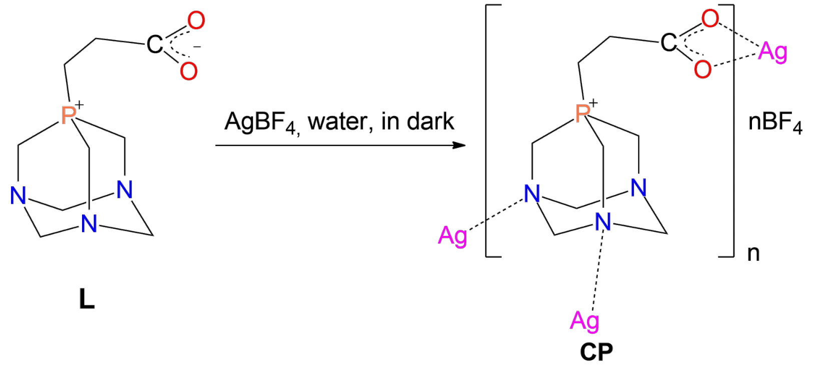

Figure 1

[Schematic diagram of formation of the title compound.]

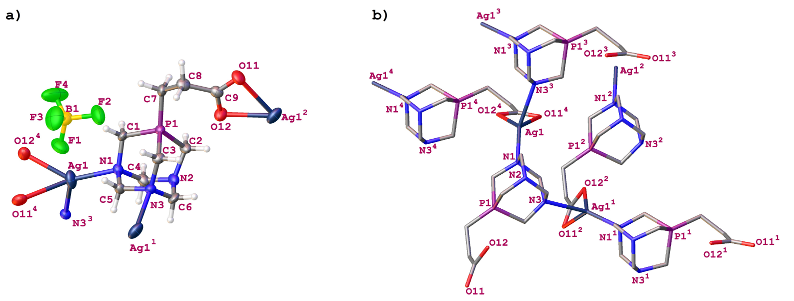

Figure 2

[(a) A view of the **CP** with the atomic labels. The atomic displacement ellipsoids are drawn at 50% probability level. (b) The coordination architecture of the **CP** with the atomic labels of the coordination sphere. Hydrogen atoms and BF_4^--ions are omitted for clarity. (Symmetry codes: ^{1}+x,-y,1/2+z; ^{2}-1/2+x,-1/2-y,1/2+z; ^{3}+x,-y,-1/2+z; ^{4}1/2+x,-1/2-y,-1/2+z).]

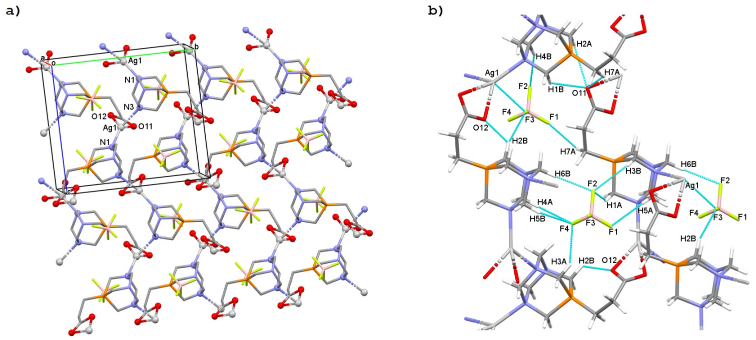

Figure 3

[(a) Packing arrangement of the three-dimensional structure of the **CP** crystal viewed from the crystallographic "***a***" axes. The coordination sphere is labelled and highlighted by a ball and stick model. Hydrogen atoms are omitted for clarity. (b) Selected hydrogen bond geometry in **CP** showing the weak C–H···F and C–H···O secondary interactions, as well as the Ag1-F3 interaction. For symmetry codes see Table 1.]

supporting information

Novel three-dimensional coordination polymer of 7-(2-carboxy-ethyl)-1,3,5-tri–aza-7-(phosphoniatricyclo)[3.3.1.1^{3,7}]decane with silver(I)-tetraflouroborate

Antal Udvardy, Agnes Katho, Gabor Papp, Ferenc Joo and Gyula Tamas Gal*

Computing details

Data collection: MACH3/PC (Enraf Nonius, 1992); cell refinement: MACH3/PC (Enraf Nonius, 1992); data reduction: PROFIT (Streltsov & Zavodnik, 1989); program(s) used to solve structure: *SIR97* (Burla *et al.*, 2007); program(s) used to refine structure: *SHELXL* (Sheldrick, 2015); molecular graphics: Olex2 1.3 (Dolomanov *et al.*, 2009); software used to prepare material for publication: Olex2 1.3 (Dolomanov *et al.*, 2009), *publCIF* (Westrip, 2010).

(CP)

Crystal data

$C_9H_{16}AgN_3O_2P\cdot BF_4$
M_r = 423.90
Monoclinic, *Cc*
a = 10.116 (5) Å
b = 12.186 (5) Å
c = 10.979 (5) Å
β = 98.260 (5)°
V = 1339.4 (11) Å^3
Z = 4
$F(000)$ = 840
D_x = 2.102 Mg m^{-3}
Mo $K\alpha$ radiation, λ = 0.71073 Å
Cell parameters from 25 reflections
θ = 9.1–17.2°
μ = 1.68 mm^{-1}
T = 293 K
Prism, colourless
0.35 × 0.2 × 0.15 mm

Data collection

Enraf Nonius CAD4 diffractometer
profiled ω/2θ scans
Absorption correction: ψ scan
North A.C.T., Phillips D.C. & Mathews F.S. (1968) Acta. Cryst. A24, 351-359 Number of ψ scan sets used was 3 Theta correction was applied. Averaged transmission function was used. Fourier smoothing - Window value 5
T_{min} = 0.558, T_{max} = 0.755
1358 measured reflections
1313 independent reflections
1299 reflections with $I > 2\sigma(I)$
R_{int} = 0.009
θ_{max} = 25.5°, θ_{min} = 3.1°
h = 0→12
k = 0→14
l = −13→13
3 standard reflections every 184 reflections
intensity decay: 2%

Refinement

Refinement on F^2
Least-squares matrix: full
$R[F^2 > 2\sigma(F^2)]$ = 0.048
$wR(F^2)$ = 0.123
S = 1.13
1313 reflections
190 parameters
2 restraints
Primary atom site location: structure-invariant direct methods
Hydrogen site location: inferred from neighbouring sites
H-atom parameters constrained
$w = 1/[\sigma^2(F_o^2) + (0.0873P)^2 + 4.2725P]$ where $P = (F_o^2 + 2F_c^2)/3$
$(\Delta/\sigma)_{max}$ < 0.001
$\Delta\rho_{max}$ = 1.20 e Å^{-3}
$\Delta\rho_{min}$ = −1.54 e Å^{-3}
Absolute structure: Classical Flack method preferred over Parsons because s.u. lower.
Absolute structure parameter: 0.13 (6)

Special details

Geometry. All esds (except the esd in the dihedral angle between two l.s. planes) are estimated using the full covariance matrix. The cell esds are taken into account individually in the estimation of esds in distances, angles and torsion angles; correlations between esds in cell parameters are only used when they are defined by crystal symmetry. An approximate (isotropic) treatment of cell esds is used for estimating esds involving l.s. planes.

Fractional atomic coordinates and isotropic or equivalent isotropic displacement parameters (Å^2) for (CP)

	x	y	z	U_{iso}*/U_{eq}
C1	−0.0444 (9)	−0.2414 (7)	−0.2977 (8)	0.0266 (16)
H1A	0.0509	−0.2557	−0.2849	0.032*
H1B	−0.0820	−0.2737	−0.3758	0.032*
C2	−0.2914 (9)	−0.2548 (7)	−0.2148 (9)	0.0302 (19)
H2A	−0.3323	−0.2870	−0.2918	0.036*
H2B	−0.3444	−0.2743	−0.1511	0.036*
C3	−0.0546 (9)	−0.2182 (7)	−0.0445 (8)	0.0284 (17)
H3A	−0.0975	−0.2365	0.0265	0.034*
H3B	0.0408	−0.2298	−0.0232	0.034*
C4	−0.2157 (10)	−0.1011 (7)	−0.3251 (9)	0.0317 (19)
H4A	−0.2311	−0.0233	−0.3392	0.038*
H4B	−0.2532	−0.1397	−0.3994	0.038*
C5	−0.0139 (9)	−0.0704 (7)	−0.1840 (8)	0.0269 (16)
H5A	0.0798	−0.0897	−0.1646	0.032*
H5B	−0.0192	0.0087	−0.1934	0.032*
C6	−0.2256 (10)	−0.0841 (8)	−0.1135 (10)	0.035 (2)
H6A	−0.2706	−0.1113	−0.0472	0.042*
H6B	−0.2418	−0.0057	−0.1203	0.042*
C7	−0.0690 (9)	−0.4434 (7)	−0.1593 (9)	0.0311 (19)
H7A	−0.1281	−0.4868	−0.2178	0.037*
H7B	0.0201	−0.4492	−0.1815	0.037*
C8	−0.0676 (11)	−0.4934 (8)	−0.0308 (9)	0.0340 (19)
H8A	−0.0322	−0.5674	−0.0306	0.041*
H8B	−0.0084	−0.4506	0.0285	0.041*
C9	−0.2032 (10)	−0.4967 (8)	0.0078 (8)	0.0298 (18)
N1	−0.0681 (7)	−0.1216 (6)	−0.3016 (6)	0.0253 (14)
N2	−0.2837 (7)	−0.1355 (6)	−0.2263 (8)	0.0323 (17)
N3	−0.0822 (8)	−0.1022 (6)	−0.0806 (7)	0.0278 (15)
O11	−0.2377 (9)	−0.5691 (6)	0.0750 (8)	0.0444 (17)
O12	−0.2793 (8)	−0.4175 (6)	−0.0319 (7)	0.0408 (16)
P1	−0.1202 (2)	−0.30412 (17)	−0.1746 (2)	0.0234 (4)
B1	0.3222 (13)	−0.2721 (10)	−0.1875 (11)	0.039 (2)
Ag1	0.03866 (7)	−0.03737 (7)	−0.43859 (7)	0.0488 (3)
F1	0.2885 (9)	−0.1691 (6)	−0.2226 (8)	0.062 (2)
F2	0.2229 (8)	−0.3155 (7)	−0.1250 (9)	0.066 (2)
F3	0.4361 (8)	−0.2745 (9)	−0.1032 (10)	0.082 (3)
F4	0.3319 (16)	−0.3351 (7)	−0.2846 (9)	0.104 (4)

Atomic displacement parameters (Å^2) for (CP)

	U^{11}	U^{22}	U^{33}	U^{12}	U^{13}	U^{23}
C1	0.025 (4)	0.023 (4)	0.033 (4)	0.002 (3)	0.013 (3)	−0.004 (3)

C2	0.022 (4)	0.022 (4)	0.047 (5)	0.005 (3)	0.006 (4)	0.005 (4)
C3	0.029 (4)	0.027 (4)	0.029 (4)	0.007 (3)	0.003 (3)	0.001 (3)
C4	0.028 (5)	0.027 (4)	0.040 (5)	−0.001 (3)	0.001 (4)	0.008 (3)
C5	0.029 (4)	0.025 (4)	0.027 (4)	−0.004 (3)	0.008 (3)	−0.003 (3)
C6	0.031 (5)	0.028 (4)	0.049 (5)	0.010 (4)	0.019 (4)	−0.001 (4)
C7	0.026 (4)	0.029 (4)	0.042 (5)	−0.001 (3)	0.016 (4)	0.000 (4)
C8	0.040 (5)	0.031 (4)	0.030 (4)	0.004 (4)	0.001 (4)	0.009 (4)
C9	0.033 (5)	0.026 (4)	0.028 (4)	−0.001 (4)	−0.002 (3)	0.002 (4)
N1	0.023 (3)	0.025 (3)	0.027 (3)	−0.001 (3)	0.003 (3)	0.005 (3)
N2	0.023 (4)	0.027 (4)	0.048 (5)	0.001 (3)	0.008 (3)	0.005 (3)
N3	0.030 (4)	0.021 (3)	0.033 (4)	0.001 (3)	0.009 (3)	−0.002 (3)
O11	0.049 (4)	0.034 (3)	0.052 (4)	−0.002 (3)	0.013 (3)	0.016 (3)
O12	0.042 (4)	0.037 (3)	0.046 (4)	0.011 (3)	0.016 (3)	0.012 (3)
P1	0.0211 (10)	0.0193 (9)	0.0306 (10)	0.0024 (8)	0.0065 (8)	0.0009 (8)
B1	0.043 (7)	0.038 (6)	0.037 (5)	0.009 (5)	0.012 (5)	0.008 (5)
Ag1	0.0465 (4)	0.0623 (5)	0.0407 (4)	−0.0149 (4)	0.0167 (3)	0.0099 (4)
F1	0.059 (4)	0.037 (3)	0.090 (6)	0.000 (3)	0.007 (4)	0.016 (3)
F2	0.040 (4)	0.064 (5)	0.096 (6)	−0.004 (3)	0.016 (4)	0.024 (4)
F3	0.038 (4)	0.114 (8)	0.091 (6)	0.013 (5)	−0.003 (4)	0.020 (6)
F4	0.205 (14)	0.049 (4)	0.065 (5)	0.025 (6)	0.047 (7)	−0.003 (4)

Geometric parameters (Å, º) for (CP)

C1—H1A	0.9700	C6—N2	1.436 (14)
C1—H1B	0.9700	C6—N3	1.460 (12)
C1—N1	1.479 (11)	C7—H7A	0.9700
C1—P1	1.815 (9)	C7—H7B	0.9700
C2—H2A	0.9700	C7—C8	1.534 (13)
C2—H2B	0.9700	C7—P1	1.775 (9)
C2—N2	1.461 (11)	C8—H8A	0.9700
C2—P1	1.826 (9)	C8—H8B	0.9700
C3—H3A	0.9700	C8—C9	1.494 (15)
C3—H3B	0.9700	C9—O11	1.233 (12)
C3—N3	1.484 (11)	C9—O12	1.272 (12)
C3—P1	1.818 (9)	N1—Ag1	2.225 (7)
C4—H4A	0.9700	N3—Ag1^{i}	2.505 (7)
C4—H4B	0.9700	O11—Ag1^{ii}	2.594 (9)
C4—N1	1.499 (12)	O12—Ag1^{ii}	2.298 (8)
C4—N2	1.428 (13)	B1—F1	1.343 (14)
C5—H5A	0.9700	B1—F2	1.399 (14)
C5—H5B	0.9700	B1—F3	1.370 (15)
C5—N1	1.467 (10)	B1—F4	1.328 (16)
C5—N3	1.464 (12)	Ag1—N3^{iii}	2.505 (7)
C6—H6A	0.9700	Ag1—O11^{iv}	2.594 (9)
C6—H6B	0.9700	Ag1—O12^{iv}	2.298 (8)
H1A—C1—H1B	108.1	C7—C8—H8B	109.1
N1—C1—H1A	109.5	H8A—C8—H8B	107.8
N1—C1—H1B	109.5	C9—C8—C7	112.6 (8)
N1—C1—P1	110.7 (6)	C9—C8—H8A	109.1
P1—C1—H1A	109.5	C9—C8—H8B	109.1

P1—C1—H1B	109.5	O11—C9—C8	122.7 (9)
H2A—C2—H2B	108.6	O11—C9—O12	122.6 (10)
N2—C2—H2A	110.4	O12—C9—C8	114.7 (8)
N2—C2—H2B	110.4	C1—N1—C4	108.8 (7)
N2—C2—P1	106.7 (6)	C1—N1—Ag1	112.5 (5)
P1—C2—H2A	110.4	C4—N1—Ag1	112.0 (5)
P1—C2—H2B	110.4	C5—N1—C1	110.9 (6)
H3A—C3—H3B	108.5	C5—N1—C4	108.6 (7)
N3—C3—H3A	110.2	C5—N1—Ag1	104.0 (5)
N3—C3—H3B	110.2	C4—N2—C2	113.3 (7)
N3—C3—P1	107.8 (6)	C4—N2—C6	110.2 (8)
P1—C3—H3A	110.2	C6—N2—C2	112.3 (8)
P1—C3—H3B	110.2	C3—N3—Ag1^{i}	115.1 (5)
H4A—C4—H4B	107.7	C5—N3—C3	111.6 (7)
N1—C4—H4A	108.9	C5—N3—Ag1^{i}	93.4 (5)
N1—C4—H4B	108.9	C6—N3—C3	110.6 (7)
N2—C4—H4A	108.9	C6—N3—C5	109.4 (7)
N2—C4—H4B	108.9	C6—N3—Ag1^{i}	115.4 (5)
N2—C4—N1	113.4 (7)	C9—O11—Ag1^{ii}	85.8 (6)
H5A—C5—H5B	107.6	C9—O12—Ag1^{ii}	98.7 (6)
N1—C5—H5A	108.7	C1—P1—C2	99.7 (4)
N1—C5—H5B	108.7	C1—P1—C3	101.4 (4)
N3—C5—H5A	108.7	C3—P1—C2	103.1 (4)
N3—C5—H5B	108.7	C7—P1—C1	108.9 (4)
N3—C5—N1	114.2 (7)	C7—P1—C2	126.3 (4)
H6A—C6—H6B	107.6	C7—P1—C3	114.0 (4)
N2—C6—H6A	108.6	F1—B1—F2	108.8 (9)
N2—C6—H6B	108.6	F1—B1—F3	111.6 (11)
N2—C6—N3	114.6 (7)	F3—B1—F2	104.7 (9)
N3—C6—H6A	108.6	F4—B1—F1	110.8 (10)
N3—C6—H6B	108.6	F4—B1—F2	108.4 (11)
H7A—C7—H7B	107.5	F4—B1—F3	112.2 (12)
C8—C7—H7A	108.4	N1—Ag1—N3^{iii}	119.6 (3)
C8—C7—H7B	108.4	N1—Ag1—O11^{iv}	134.1 (3)
C8—C7—P1	115.5 (7)	N1—Ag1—O12^{iv}	133.5 (3)
P1—C7—H7A	108.4	N3^{iii}—Ag1—O11^{iv}	92.3 (3)
P1—C7—H7B	108.4	O12^{iv}—Ag1—N3^{iii}	103.6 (3)
C7—C8—H8A	109.1	O12^{iv}—Ag1—O11^{iv}	52.9 (2)

C7—C8—C9—O11	148.3 (10)	N2—C6—N3—C5	53.5 (10)
C7—C8—C9—O12	−33.2 (12)	N2—C6—N3—Ag1^{i}	157.2 (6)
C8—C7—P1—C1	153.3 (7)	N3—C3—P1—C1	51.8 (7)
C8—C7—P1—C2	−88.5 (8)	N3—C3—P1—C2	−51.1 (7)
C8—C7—P1—C3	40.9 (8)	N3—C3—P1—C7	168.7 (6)
C8—C9—O11—Ag1^{ii}	177.7 (9)	N3—C5—N1—C1	−66.7 (9)
C8—C9—O12—Ag1^{ii}	−177.7 (7)	N3—C5—N1—C4	52.8 (9)
N1—C1—P1—C2	54.5 (7)	N3—C5—N1—Ag1	172.2 (6)
N1—C1—P1—C3	−51.1 (7)	N3—C6—N2—C2	71.6 (10)
N1—C1—P1—C7	−171.6 (6)	N3—C6—N2—C4	−55.7 (10)
N1—C4—N2—C2	−71.1 (10)	O11—C9—O12—Ag1^{ii}	0.8 (11)
N1—C4—N2—C6	55.6 (10)	O12—C9—O11—Ag1^{ii}	−0.7 (10)

N1—C5—N3—C3	70.1 (9)	P1—C1—N1—C4	−60.8 (8)
N1—C5—N3—C6	−52.7 (9)	P1—C1—N1—C5	58.5 (8)
N1—C5—N3—Ag1^{i}	−171.2 (6)	P1—C1—N1—Ag1	174.5 (4)
N2—C2—P1—C1	−53.2 (7)	P1—C2—N2—C4	64.6 (9)
N2—C2—P1—C3	51.0 (7)	P1—C2—N2—C6	−61.0 (8)
N2—C2—P1—C7	−175.5 (6)	P1—C3—N3—C5	−62.5 (8)
N2—C4—N1—C1	66.6 (10)	P1—C3—N3—C6	59.5 (8)
N2—C4—N1—C5	−54.2 (9)	P1—C3—N3—Ag1^{i}	−167.4 (3)
N2—C4—N1—Ag1	−168.4 (6)	P1—C7—C8—C9	62.9 (10)
N2—C6—N3—C3	−69.8 (10)		

Symmetry codes: (i) *x*, −*y*, *z*+1/2; (ii) *x*−1/2, −*y*−1/2, *z*+1/2; (iii) *x*, −*y*, *z*−1/2; (iv) *x*+1/2, −*y*−1/2, *z*−1/2.

Table 1. Selected hydrogen bond distances (Å) and angles (°) in crystal CP

D—H···A	D—H	H···A	D···A	D—H···A
C1—H1A···F2	0.97	2.40	3.201 (13)	140
C1—H1B···O11$^{(i)}$	0.97	2.49	3.213 (12)	131
C2—H2A···O11$^{(i)}$	0.97	2.56	3.254 (12)	129
C2—H2B···F3$^{(ii)}$	0.97	2.35	3.183 (13)	143
C2—H2B···O12	0.97	2.22	2.812 (12)	118
C3—H3A···F4$^{(iii)}$	0.97	2.45	3.290 (15)	145
C3—H3B···F2	0.97	2.51	3.283 (12)	136
C4—H4A···F4$^{(iv)}$	0.97	2.43	3.298 (12)	148
C4—H4B···F2$^{(v)}$	0.97	2.51	3.417 (14)	155
C5—H5A···F1	0.97	2.49	3.370 (13)	151
C5—H5B···F4$^{(iv)}$	0.97	2.54	3.373 (14)	144
C6—H6B···F2$^{(iv)}$	0.97	2.34	3.314 (13)	177
C7—H7A···O11$^{(ii)}$	0.97	2.48	3.165 (13)	128
C7—H7A···F1$^{(vi)}$	0.97	2.37	3.137 (12)	135

Symmetry codes: $^{(i)}$ x, –1–y, –1/2+z; $^{(ii)}$ –1+x, y, z; $^{(iii)}$ –1/2+x, –1/2–y, 1/2+z; $^{(iv)}$ –1/2+x, 1/2+y, z; $^{(v)}$ – 1/2+x, –1/2–y, –1/2+z; $^{(vi)}$ – 1/2+x, –1/2+y, z
