# Supplementary material for: Novel three-dimensional coordination polymer of 2-(1,3,5-tri­aza-7-phospho­niatri­cyclo­[3.3.1.13,7]decan-7-yl)ethanoic acid with silver(I) tetra­fluoro­borate
Source: Acta Crystallogr E Crystallogr Commun. 2022 Feb 1;78(Pt 3):251–4. doi: 10.1107/S2056989022000767 (PMC8900499; doi:10.1107/S2056989022000767)
Supplement: Supplementary file 4 [file e-78-00251-sup4.pdf]

UNIVERSITY of DEBRECEN

UNIVERSITY OF DEBRECEN
FACULTY OF SCIENCE AND TECHNOLOGY
Department of Physical Chemistry
H-4002 Egyetem tér 1, Debrecen

December 21, 2021

Dear Prof. A. J. Allen,

It was a great honour for me to attend the 32nd European Crystallographic Meeting (ECM32) and to win one of the IUCr Journals Poster Prizes, which is an IUCr Journals Prizes Voucher. Herein, I would like to use this voucher for the present article.

Herewith we submit our manuscript entitled *"Novel three-dimensional coordination polymer of 7-(2-carboxy-ethyl)-1,3,5-triaza-7-(phosphoniatricyclo)[3.3.1.1^{3,7}]decane with silver(I)-tetraflouroborate"* (authors: *Antal Udvardy*, Ágnes Kathó, Gábor Papp, Ferenc Joó, Gyula Tamás Gál**) and ask you to consider it for publication in *Acta Crystallographica Section E*. This submission is original and is not under consideration elsewhere. All authors are aware of this submission which is made having their agreement. We declare no competing interests.

The preparation of Ag-based coordination polymers with 1,3,5-triaza-7-phosphaadamantane(PTA) or its derivatives is of great interest nowadays. In our previous article published in *Inorganic Chimica Acta*, we fully characterized three 1D coordination polymers formed from 7-(2-carboxy-ethyl)-1,3,5-triaza-7-(phosphoniatricyclo)[3.3.1.1^{3,7}]-decane (**L**) with different Ag-salts ($AgPF_6$, $AgSO_3C_6H_4CH_3$, $AgSO_3CF_3$) in water. In contrast, as described in the present paper, a novel 3D Ag-based coordination polymer was observed in the reaction of the $AgBF_4$ and **L** in aqueous solution. The title compound was also characterized by X-ray diffraction method and multinuclear NMR and HR-MS spectroscopies and elemental analysis, too.

We hope that the contents and quality of this paper match the requirements of the Journal and you will find this work timely and useful to be published in *Acta Crystallographica Section E.*

With all best regards.

Sincerely,

Gyula Tamás Gál

and

Antal Udvardy, PhD

University of Debrecen
