# Supplementary material for: Novel three-dimensional coordination polymer of 2-(1,3,5-tri­aza-7-phospho­niatri­cyclo­[3.3.1.13,7]decan-7-yl)ethanoic acid with silver(I) tetra­fluoro­borate
Source: Acta Crystallogr E Crystallogr Commun. 2022 Feb 1;78(Pt 3):251–4. doi: 10.1107/S2056989022000767 (PMC8900499; doi:10.1107/S2056989022000767)
Supplement: Supplementary file 7 [file e-78-00251-sup7.pdf]

UNIVERSITY of DEBRECEN

UNIVERSITY OF DEBRECEN
FACULTY OF SCIENCE AND TECHNOLOGY
Department of Physical Chemistry
H-4002 Egyetem tér 1, Debrecen

January 4, 2022

Dear Professor J. Ellena,

Herewith we submit the revised version of our manuscript (ex2052) entitled „Novel three-dimensional coordination polymer of 7-(2-carboxy-ethyl)-1,3,5-triaza-7-(phosphoniatricyclo)[3.3.1.1^{3,7}]decane with silver(I)-tetraflouroborate (Authors: Antal Udvardy*, Ágnes Kathó, Gábor Papp, Ferenc Joó and Gyula Tamás Gál*.
Please find below our response to your questions and comments.

Responses:

(Q1)1- The "tetrafluoroborate" needs to be corrected in all the manuscript.

AR: We carefully checked the manuscript and corrected it, thanks to the reviewer.

(Q2)2- The "three-dimensional" in parenthesis in the abstract is unnecessary.

AR: We deleted the "three-dimensional" phrase.

(Q3)3- In the abstract, the synthesized CP is mentioned as light insensitive. How was the light sensitivity of the complex evaluated?

AR: In the presence of light, the filtrated crystals of CP remained colorless.

(Q4)4- In Scheme 1, please adjust the Ag-N bond (coordinated bond)

AR: We inserted a new version of the Scheme.

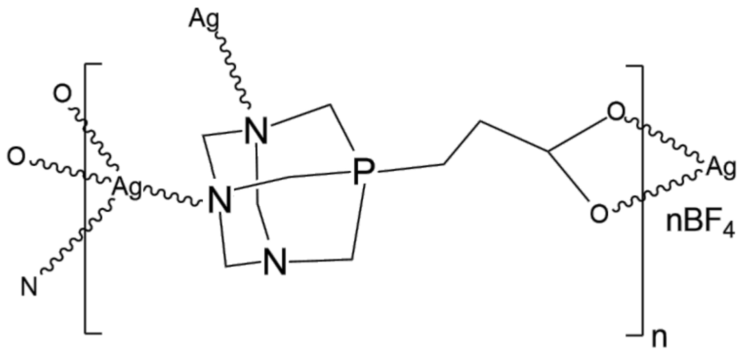

UNIVERSITY OF DEBRECEN
FACULTY OF SCIENCE AND TECHNOLOGY
Department of Physical Chemistry
H-4002 Egyetem tér 1, Debrecen

(Q5)5- It remains to put the letter "s" in hexafluorophosphate (Chemical context, third paragraph).

AR: We carefully checked the manuscript and corrected it, thanks to the reviewer.

(Q6)6- The first "N" in the description of the coordination mode of the ligand also needs to be in italic (structural commentary section).

AR: We carefully checked the manuscript and corrected it, thanks to the reviewer.

(Q7)7- To confirm the geometry of the metal center, I suggest calculating the geometry index for the complex (Okuniewski, A., Rosiak, D., Chojnacki, J. and Becker, B. (2015) Polyhedron, 90, 47).

AR: It is a very useful comment, thank you. This sentence with the references were inserted to the manuscript:
"The coordination geometry shows a distorted tetrahedral shape (τ_4=0.65 and τ_4'=0.66 [Yang *et al.*, 2007], [Okuniewski *et al.*, 2015]), in which the Ag(I) is located at the center."

References:

Okuniewski, A., Rosiak, D., Chojnacki, J. & Becker, B. (2015). *Polyhedron* **90**, 47–57.
Yang, L., Powell, D. R. & Houser, P. (2007). *Dalton Trans.* **9**, 955–964.

(Q8)8- Figure 2b is referenced to describe the BF4- counter ions occupying the voids in the structure of the coordination polymer, but the figure does not show the BF4- anions.

AR: Sorry about the mistake; we meant to refer to Fig.3b instead of Fig.2b. It was corrected.

(Q9)9- As a suggestion, I think it would be more interesting to describe a little about the importance of coordination polymers in the chemical context section.

AR: A new sentence was inserted to the manuscript:
„The architectures and antimicrobial properties of self-assembly silver-based coordination polymers or MOFs (Metal-Organic Frameworks), bridged by phosphaurotropines, have been widely studied [Guerriero *et al.*, 2018]."

Reference:

Guerriero, A., Peruzzini, H. & Gonsalvi, L. (2018). *Coord. Chem. Rev.*, **355**, 328–361.

UNIVERSITY of DEBRECEN

UNIVERSITY OF DEBRECEN
FACULTY OF SCIENCE AND TECHNOLOGY
Department of Physical Chemistry
H-4002 Egyetem tér 1, Debrecen

Overall, we feel that the revision made the manuscript better and hope that now it can be found suitable for publication in Acta Crystallographica Section E.

With all best regards.
Sincerely,

Gyula Tamás Gál
and
Antal Udvardy, PhD
University of Debrecen
